# Simmons–Smith Cyclopropanation: A Multifaceted Synthetic Protocol toward the Synthesis of Natural Products and Drugs: A Review

**DOI:** 10.3390/molecules28155651

**Published:** 2023-07-26

**Authors:** Ramsha Munir, Ameer Fawad Zahoor, Sadia Javed, Bushra Parveen, Asim Mansha, Ahmad Irfan, Samreen Gul Khan, Ali Irfan, Katarzyna Kotwica-Mojzych, Mariusz Mojzych

**Affiliations:** 1 Medicinal Chemistry Research Lab, Department of Chemistry, Government College University Faisalabad, Faisalabad 38000, Pakistan; ramshamunir2017@gmail.com (R.M.); bushrachemgcuf@gmail.com (B.P.); asimmansha@gcuf.edu.pk (A.M.); samreengul@gcuf.edu.pk (S.G.K.); raialiirfan@gmail.com (A.I.); 2 Department of Biochemistry, Government College University Faisalabad, Faisalabad 38000, Pakistan; sadiajaved@gcuf.edu.pk; 3 Department of Chemistry, College of Science, King Khalid University, Abha 61413, Saudi Arabia; irfaahmad@gmail.com; 4 Laboratory of Experimental Cytology, Medical University of Lublin, Radziwiłłowska 11, 20-080 Lublin, Poland; katarzynakotwicamojzych@umlub.pl; 5 Department of Chemistry, Siedlce University of Natural Sciences and Humanities, 3-go Maja 54, 08-110 Siedlce, Poland

**Keywords:** Simmons–Smith cyclopropanation, natural products, drugs, Furukawa reagent

## Abstract

Simmons–Smith cyclopropanation is a widely used reaction in organic synthesis for stereospecific conversion of alkenes into cyclopropane. The utility of this reaction can be realized by the fact that the cyclopropane motif is a privileged synthetic intermediate and a core structural unit of many biologically active natural compounds such as terpenoids, alkaloids, nucleosides, amino acids, fatty acids, polyketides and drugs. The modified form of Simmons–Smith cyclopropanation involves the employment of Et_2_Zn and CH_2_I_2_ (Furukawa reagent) toward the total synthesis of a variety of structurally complex natural products that possess broad range of biological activities including anticancer, antimicrobial and antiviral activities. This review aims to provide an intriguing glimpse of the Furukawa-modified Simmons–Smith cyclopropanation, within the year range of 2005 to 2022.

## 1. Introduction

**Figure 1:**
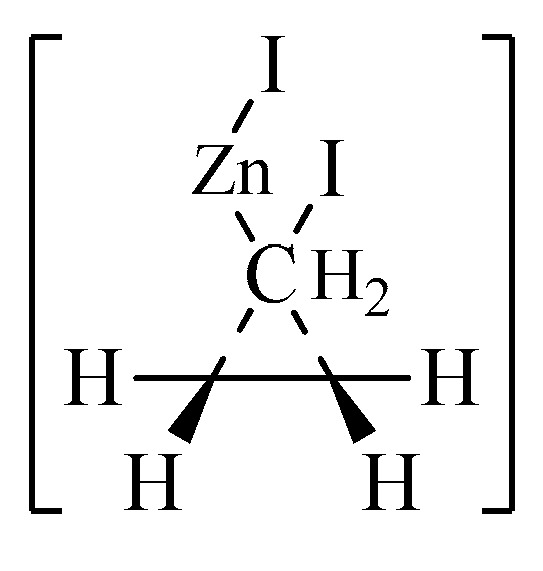
Transition state of Simmons–Smith cyclopropanation.

Simmons–Smith reagent [Et_2_Zn, CH_2_I_2_ or Zn, CH_2_I_2_=ICH_2_ZnI] is one of the metal carbenoids that has been widely used in the cyclopropanation of olefins and allylic alcohols [1,2,3,4,5]. Simmons–Smith reagent was discovered by Simmons and Smith in 1958, when they performed stereospecific synthesis of cyclopropanes in high yield by reacting alkenes with diiodomethane in the presence of zinc [6]. This zinc carbenoid reagent is a powerful synthetic tool for the stereoselective addition of a methylene unit to chiral olefins [7]. The utility of this reaction in organic chemistry is due to the high stereospecific nature and efficient chiral version (>90% ee) that avoids the use of covalently bonded chiral auxiliaries [3,7,8,9]. More particularly, Simmons–Smith cyclopropanation is a well-suited protocol for the conversion of cationically polymerizable olefins (such as vinyl ethers) into the corresponding cyclopropanes [4,8]. The enantiopure synthesis of various allylic alcohols has been reported in the literature by employing asymmetric Simmons–Smith cyclopropanation [3]. The mechanistic studies of Simmons–Smith cyclopropanation postulated that iodomethylzinc iodide and alkene reacts to form a butterfly-shaped transition state and proceeds in a concerted fashion to produce cyclopropanes (Figure 1) [2,6,7,8].

The rate of the Simmons–Smith cyclopropanation reaction depends upon various factors such as solvent [7], substituents present on the substrate [8,9,10,11] and the nature of substituent present on the zinc carbenoid [8,12,13,14]. The choice of solvent in this reaction has an important role because of the electrophilic nature of zinc carbenoid and the Lewis acidity of the reagent. The rate of Simmons–Smith cyclopropanation decreases as the basicity of the solvent increases. The generally used solvents for this reaction include dichloromethane, 1,2-dichloroethane [7] and cyclopentyl methyl ether (CPME) [15], as these are non-basic and unreactive toward zinc reagent. Furthermore, these are polar enough to stabilize the substrates [7]. The presence of various heteroatoms in substrates (acting as a directing group) increases the rate of reaction by creating an orderly transition state to achieve an effective stereocontrol. The electron-rich olefins react faster with carbenoids than those of electron-poor olefins. A variety of chiral auxiliaries/functionalities, namely, ketals, allylic ethers, enol ethers, chiral enamides and vinyl boronic esters, are well compatible toward the asymmetric Simmons–Smith cyclopropanation [2,8,9,10,11,16].

**Figure 2:**
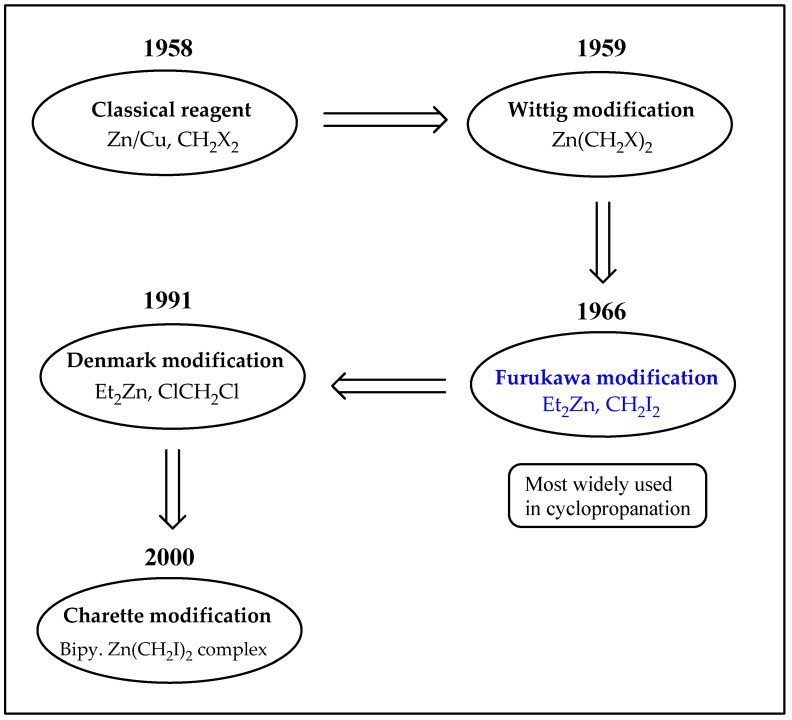
Various modifications for Simmons–Smith cyclopropanation [9].

Initially, only CH_2_I_2_ and Zn-Cu were used for cyclopropanation but were not much reactive, although they were a stable reagent. With the passage of time, various modifications have been made in this reagent [6,7]. Wittig modified the original Simmons–Smith reagent by reacting ZnX_2_ with CH_2_N_2_ to form Zn(CH_2_X)_2_. In 1966, the Furukawa modification was performed by reacting Et_2_Zn and CH_2_I_2_ while performing cyclopropanation on a series of polymerizable olefins. The syntheses of bicyclo[4.1.0]heptane and many other cyclopropanated products have been reported by using this methodology [8]. Denmark disclosed that the chloro-substituted reagent (generated from Et_2_Zn and ClCH_2_I) is more reactive than the Iodo-substituted one (Et_2_Zn and CH_2_I_2_) [16]. According to Charette, bipy. Zn(CH_2_I)_2_ complex is very efficient, as it can be isolated easily and stored in a freezer for a long time [3]. In the continuation of this work, several (halomethyl)zinc compounds and their complexes have been studied by Denmark and Charette [3,16]. The use of acidic additives such as substituted phenols and CF_3_CO_2_H, in addition to Et_2_Zn and CH_2_I_2_, is also considered efficient, especially for the cyclopropanation of less reactive alkenes (Figure 2).

**Figure 3:**
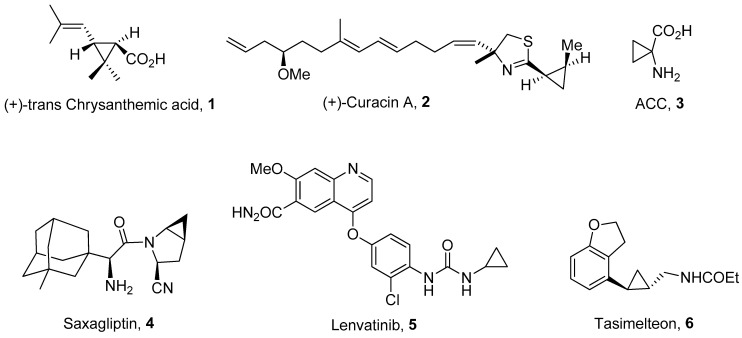
Some biologically active natural compounds.

The cyclopropyl unit exists as a core structural unit in a large family of natural and biologically active compounds such as alkaloids, terpenoids, amino acids, nucleosides, polyketides, fatty acids and drugs. These cyclopropane rings containing natural compounds exhibit a remarkable pharmaceutical profile and a broad range of biological activities including antifungal, antiviral, anti-inflammatory, antihypertensive, anticancer, antibiotic and antibacterial activities [10,11,17,18]. Simmons–Smith cyclopropanation is well suited for the diastereoselective and enantioselective synthesis of these natural products with the exact incorporation of desired stereogenic centers. (+)-Trans-chrysanthemic acid **1**, (+)-curacin A **2**, 1-aminocyclopropanecarboxylic acid (ACC) **3**, saxagliptin **4**, lenvatinib **5** and tasimelteon **6** are examples of some biologically active natural products, whose total synthesis involve Simmons–Smith cyclopropanation as the main step. The structures of these heterocyclic scaffolds are given below (Figure 3) [19,20,21,22,23].

Moreover, Simmons–Smith reagent is also involved in the efficient asymmetric cyclopropanations of various other heterocyclic scaffolds, such as bicyclic olefins (e.g., bicyclo [2.2.l] heptene and bicyclo [2.2.ll] heptadiene) [8] and a variety of chiral auxiliaries including chiral ketals, allylic alcohols, allylic ethers, enol ethers, chiral enamides and vinyl boronic esters [3,24,25]. However, in this review, we have summarized the scope of the Furukawa variant of Simmons–Smith cyclopropanation toward the synthesis of natural products and some drugs.

## 2. Review of Literature

### 2.1. Synthesis of Alkaloids Based Natural Products 

#### 2.1.1. Bisindole Alkaloids

**Scheme 1:**
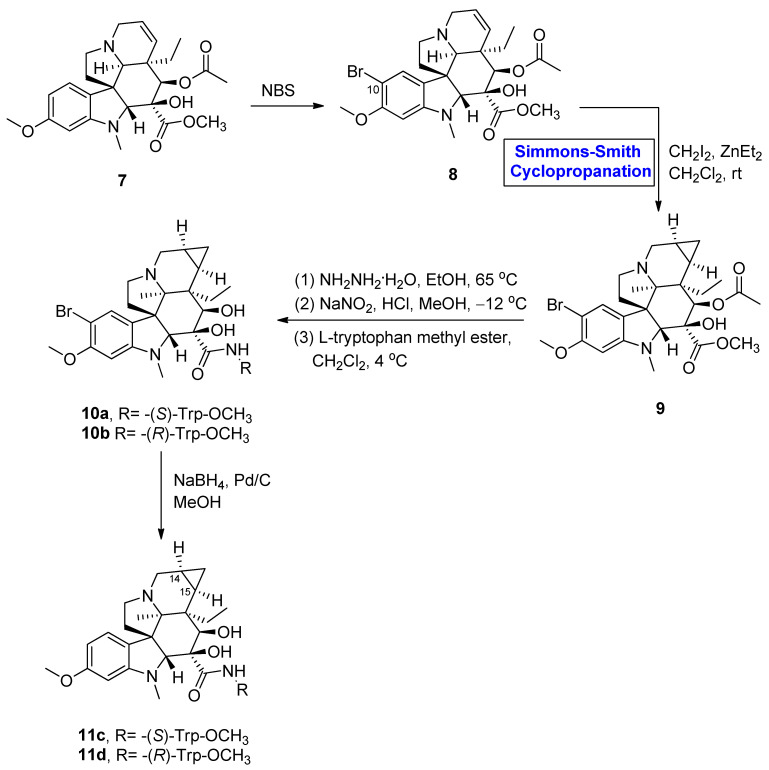
Synthesis of amino acid derivative of vindoline.

Vinblastine and vincristine (former derivative of vinblastine) belong to the class of “bisindole” alkaloids, also known as dimeric alkaloids. These are isolated from the Madagascar periwinkle plant *Catharanthus roseus*. Two monomers of these alkaloids are vindoline and catharanthine. Both vinblastine and vincistine are anti-microtubule drugs, used in the treatment of various kinds of cancer, which is a leading cause of death worldwide [26,27,28]. Vinblastine is used for the treatment of head and neck cancer, breast cancer, testicular cancer and Hodgkin’s lymphoma. Vincristine is specialized for the treatment of acute lymphoblastic leukemia, Hodgkin’s and non-Hodgkin’s lymphoma and nephroblastoma [29]. The attractive pharmacological profile of these two dimeric alkaloids prompted researchers to synthesize their derivatives and evaluate their biological activities. Keglevich et al. in 2015 synthesized amino acid derivatives of both vinblastine and vincristine by coupling a cyclopropanated (C14 and C15 position) vindoline part with (D)- and (L)-tryptophan methyl esters (at C16 position) [30]. In their synthetic route, vindoline **7** was brominated by treating with NBS to produce bromovindoline **8**. Compound **8** was then cyclopropanated in a stereospecific manner by using Simmons–Smith reagent, i.e., diethylzinc, diiodomethane and dichloromethane at 0 °C, and then raising the temperature up to 25 °C, which furnished vindoline derivative **9** with a successfully installed cyclopropane ring. Compound **9** was then treated with N_2_H_4_^.^H_2_O in the presence of EtOH to form hydrazide, followed by azide formation by using NaNO_2_ and HCl in methanol to produce an intermediate, which was proceeded further for coupling with tryptophan methyl ester at 4 °C to afford compounds **10a** and **10b**, which on subsequent reduction resulted in **11a** and **11b**. The antitumor activities of the synthesized derivatives (**10a**, **10b**, **11a** and **11b**) were evaluated in vitro against an HL-60 human leukemic cell line by MIT assay. The IC_50_ values of **10a**, **10b**, **11a** and **11b** against this cell line were 75.3 μm, 72.6 μm, 77.1 μm and >100 μm, respectively (Scheme 1).

**Scheme 2:**
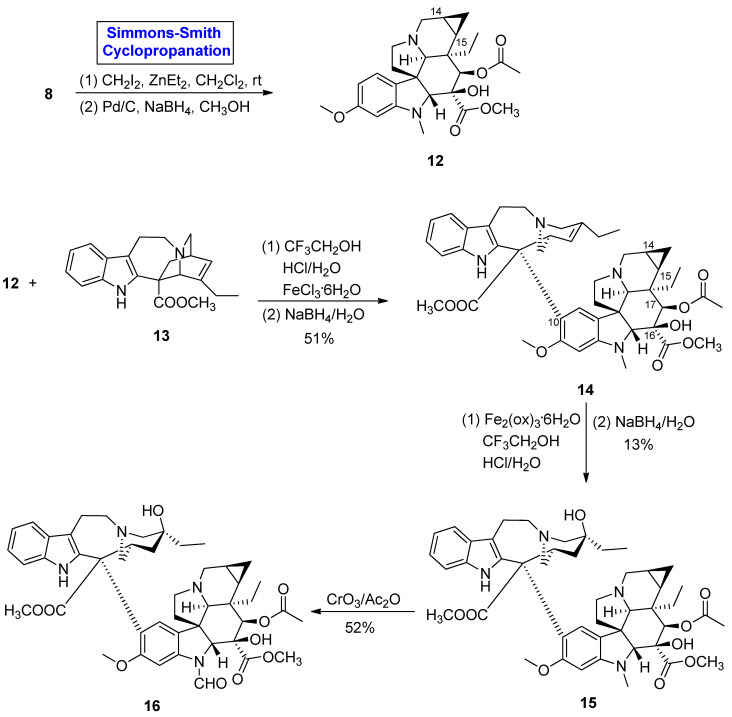
Synthesis of 14,15-cyclopropanaovinblastine **15** and 14,15-cyclopropanovincristine **16**.

In another route, the cyclopropanated derivative **9** was reduced by using a palladium catalyst in the presence of sodium borohydrate to produce 14,15-cyclopropanovindoline **12**. Compound **12** was then allowed to couple with catharanthine **13** to produce compound **14** in a 51% yield [30]. The treatment of compound **14** with oxalate salt and Fe_2_(ox)_3_ resulted in 14,15-cyclopropanaovinblastine **15**, which after chromatographic purification produced a 13% yield. The oxidation of compound **15** by using chromium oxide furnished 14,15-cyclopropanovincristine **16** in a 52% yield (Scheme 2). Compounds **15** and **16** were evaluated against 56 different cancer cell lines. Compound **15** showed the best inhibiting effects in the cases of colon cancer, lung cancer, breast cancer, melanoma and leukemia. Compound **16** showed better results against melanoma, prostate cancer, colon cancer and ovarian cancer cell lines.

**Scheme 3:**
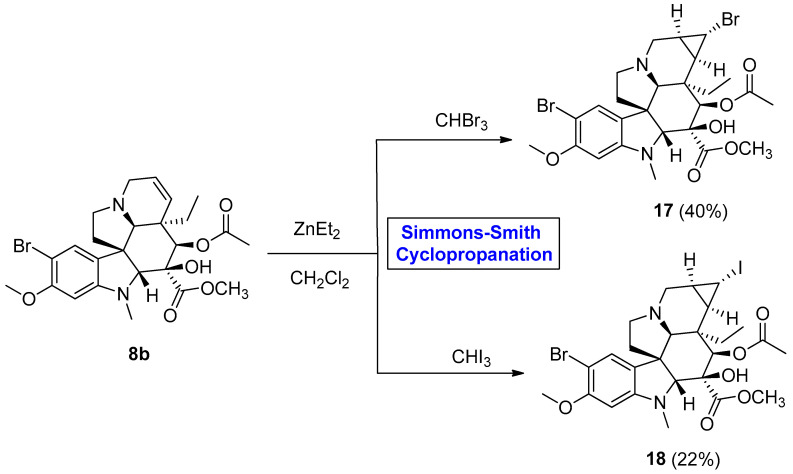
Synthesis of halogenated cyclopropanovindoline derivatives 14,15-bromocyclopropanovindoline **17** and 14,15-iodocyclopropanovindoline **18**.

In 2018, Keglevich et al., as a continuation of their work, accomplished the stereospecific synthesis of halogenated cyclopropanovindoline derivatives by using the Simmons–Smith protocol [31]. In this synthesis, vindoline derivative **8b** was allowed to react with bromoform and iodoform in the presence of diethyl zinc and dichloromethane to furnish 14,15-bromocyclopropanovindoline **17** and 14,15-iodocyclopropanovindoline **18** in 40% and 22% yields, respectively (Scheme 3).

#### 2.1.2. Kopsia Alkaloids

**Scheme 4:**
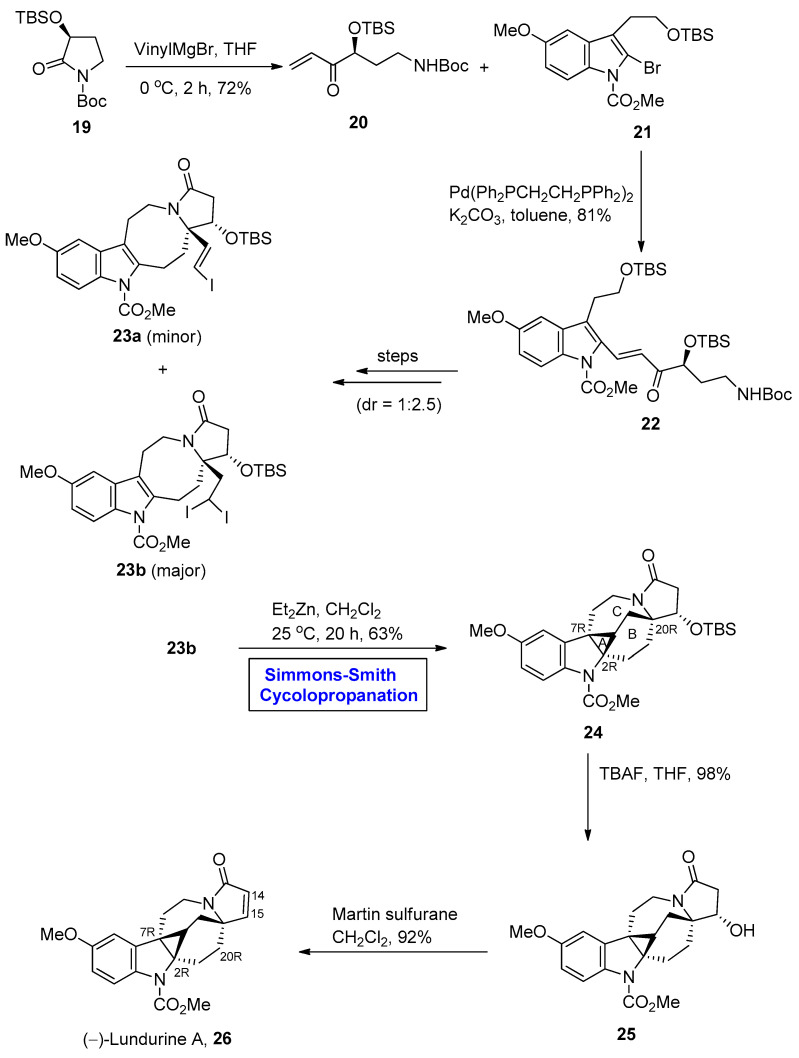
Total synthesis of (−)-lundurine A.

Lundurines A-D belongs to the class of Kopsia alkaloids. These are isolated from the plants of *Kopsia tenuis*, which are found in Malaysia [32]. These show structural similarity with indoline alkaloids. The unique molecular architecture of lundurines consists of a hexacyclic ring, an indole ring, an assembly of a three-membered ring (A), a six-membered ring (B) and a seven-membered ring (C) with three stereodefined quaternary centers. Lundurines are effective against the KB cell line with IC_50_ = 4.6–14.2 µg mL^−1^ [33]. This attractive heterocyclic scaffold has been the synthetic target of various researchers. Intensive attempts involving the synthesis of lundurine have been reported in the literature; however, none of the synthetic strategies provided lundurine with an absolute configuration of three quaternary stereocenters. Pioneering in this work, Jin et al. in 2014 disclosed the synthetic route toward the efficient and concise synthesis of (−)-lundurine A **26** in a 15-step sequence with a 2% overall yield [34]. The key step in their synthetic route involved Simmons–Smith cyclopropanation, which carefully controlled the stereochemistry at the C2 and C7 positions with simultaneous formation of ring B (six-membered) and ring C (seven-membered). In the first step, the easily available starting material (*S*)-pyrrolidinone **19** was treated with vinyl magnesium bromide to produce enone **20** in a 72% yield. The enone **20** was then allowed to react with bromo indole **21** under Heck conditions via 4 h reflux to furnish compound **22**. The modification of compound **22** in a few steps resulted in a mixture of products **23a** and **23b** (dr = 1:2.5), among which compound **23b** (as a major product after chromatographic separation) was made to react with diethyl zinc and dichloromethane at 25 °C for 20 h via Simmons–Smith cyclopropanation to furnish compound **24** (with a successfully installed cyclopropane ring) in a 63% yield. As the Simmons–Smith reaction proceeded from the upper side of the double bond in the indole ring, the configuration of C2 and C7 was deduced as 2*R* and 7*R*, respectively. In the next step, deprotection of compound **24** in the presence of TBAF and THF generated compound **25**, followed by subsequent treatment with Martin sulfurane in dichloromethane as a solvent, successfully resulted in the synthesis of our desired (−)-lundurine A **26** with a double bond at the C14 and C15 positions (Scheme 4).

#### 2.1.3. Limonoid Alkaloids

**Scheme 5:**
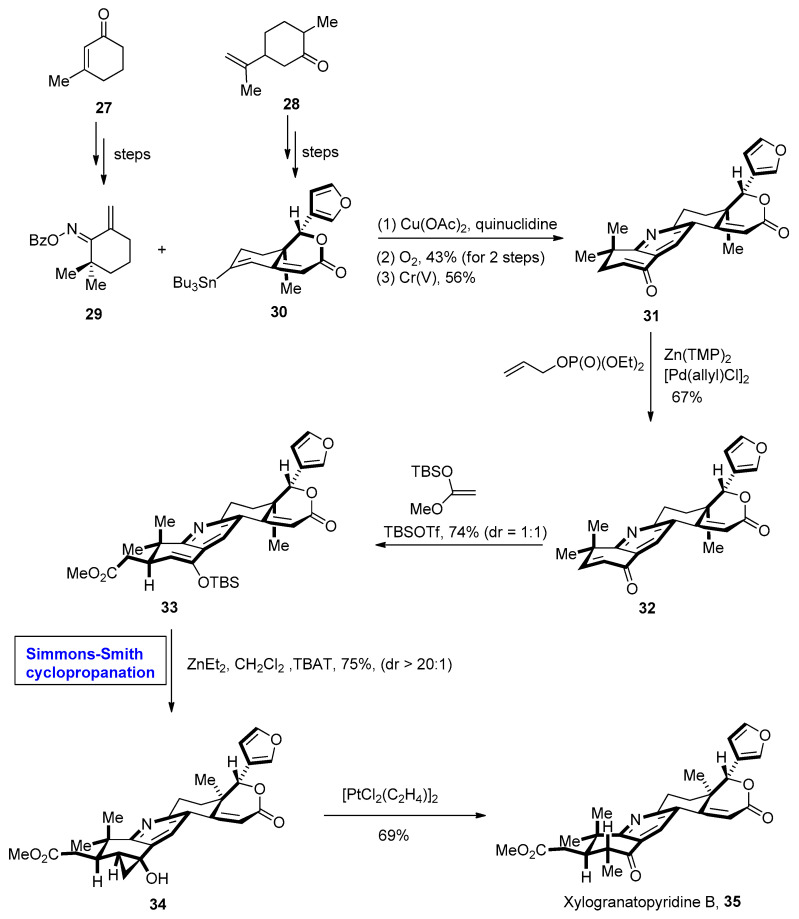
Synthesis of xylogranatopyridine B **35**.

Xylogranatopyridine B **35** belongs to the class of limonoid alkaloids. These are isolated from the leaves of *Xylocarpus granatum*, found in China [35]. These contain a pyridine ring incorporated in their basic skeleton. This sophisticated heterocyclic scaffold shows fascinating biological activities, among which their role as phosphatase inhibitor is important [36]. Keeping in view the importance of this natural product, Schuppe et al. in 2018 adopted a biomimetic strategy (based on the synthesis of Liebeskind pyridine) toward the synthesis of xylogranatopyridine B **35** and performed its total synthesis in 11 steps from a commercially available and inexpensive starting material such as dihydrocarvone [37]. The key steps entailed a Chan–Lam coupling reaction, benzylic oxidation, a Mukaiyama−Michael reaction and Simmons–Smith cyclopropanation. The oxime **29** (synthesized from 3-methyl-2-cyclohexenone **27**) and stannane fragment **30** (synthesized from dihydrocarvone **28**) were allowed to react in the Chan–Lam coupling conditions (Cu(OAc)_2_ and quinuclidine) [38], followed by selective oxidation supported by the addition of Cr(V) complex, which generated ketone **31** in a 56% yield. The *α*,*β*-dehydrogenation of ketone **31**, on 1 g scale produced intermediate **32** with a 67% yield. Furthermore, the Mukaiyama−Michael reaction of compound **32** afforded compound **33**. The next step required selective methylation, for which Simmons–Smith cyclopropanation protocol is well suited. Thus, treating **33** with diethylzinc and CH_2_I_2_ with simultaneous deprotection of siloxycyclopropane in the presence of TBSOTf resulted in compound **34**, with high diastereoselectivity (>20:1 dr), in a 74% yield. In the last step, compound **34** was treated with Zeise’s dimer, i.e., [PtCl_2_(C_2_H_4_)]_2_ furnished xylogranatopyridine B **35** in a 69% yield (Scheme 5).

#### 2.1.4. Daphniphyllum Alkaloids

**Scheme 6:**
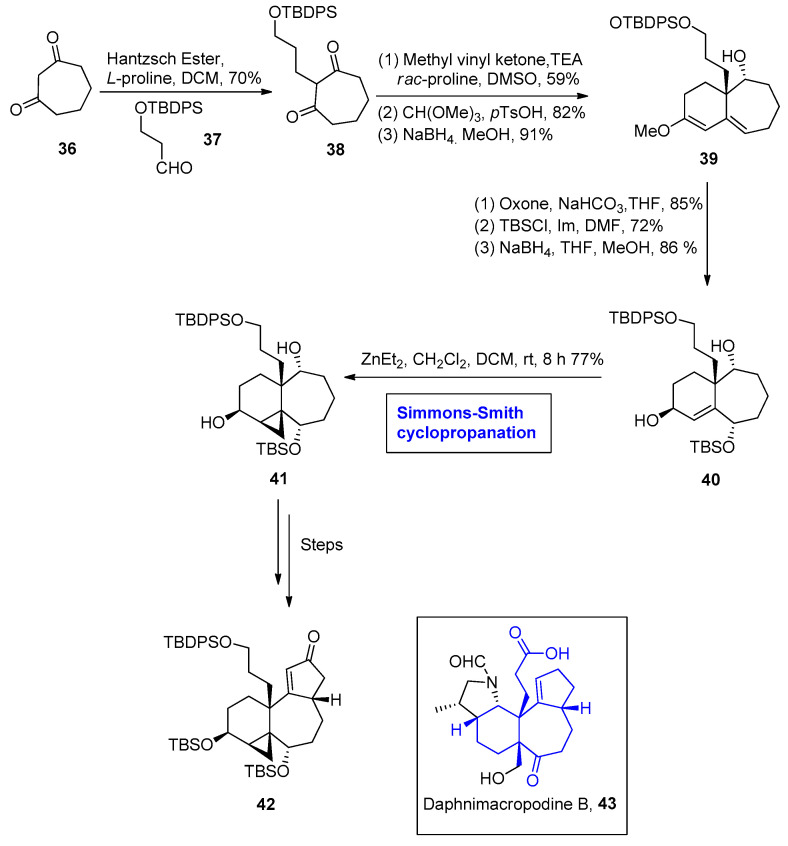
Synthesis of tricyclic skeleton of daphnimacropodine B **43**.

Daphniphyllum alkaloids are isolated from genus *Daphniphyllum*. These alkaloids are famous for exhibiting anti-HIV activities and are further classified into more than 35 sub-families. Daphnimacropodines A–C belong to the daphniglaucin-C-type sub-family of the daphniphyllum alkaloids. Their dynamic structure is based on a tetracyclic ring system with two vicinal quaternary stereodefined centers [39]. As part of the ongoing research on antiviral studies, various groups of researchers including Gao [40] and Hanessian [41] attempted the total synthesis of daphnimacropodines. Chen et al. in 2021 also performed the total synthesis of the tricyclic core skeleton of daphnimacropodine B **43** [42]. The key steps in their synthetic route entailed Robinson annulation, Simmons–Smith cyclopropanation and Horner–Wadsworth–Emmons (HWE) reaction. In their synthetic route, 1,3-cycloheptanedione **36** was allowed to react with aldehyde **37** in the presence of Hantzch ester, L-proline and DCM to produce compound **38**. The reaction of compound **38** with methyl vinyl ketone and subsequent treatment with proline (to achieve maximum enantioselectivity) produced an intermediate, which was proceeded further for reaction with CH(OMe)_3_, and subsequent reduction resulted in compound **39**. Compound **39** was oxygenated by using oxone and, after suitable protection, produced compound **40** in an excellent yield. In the next step, compound **40** underwent Simmons–Smith cyclopropanation in the presence of CH_2_I_2_, Et_2_Zn and DCM acting as a solvent, resulting in bicyclic intermediate **41** (in a 77% yield) with successful installation of two vicinal quaternary stereocenters. Intermediate **41** was then made to undergo a few steps to complete the total synthesis of our desired tricyclic core skeleton **42** of daphnimacropodine B **43** in a 62% yield (Scheme 6).

### 2.2. Synthesis of Terpenoid-Based Natural Products

#### 2.2.1. Indole Terpenoids

**Scheme 7:**
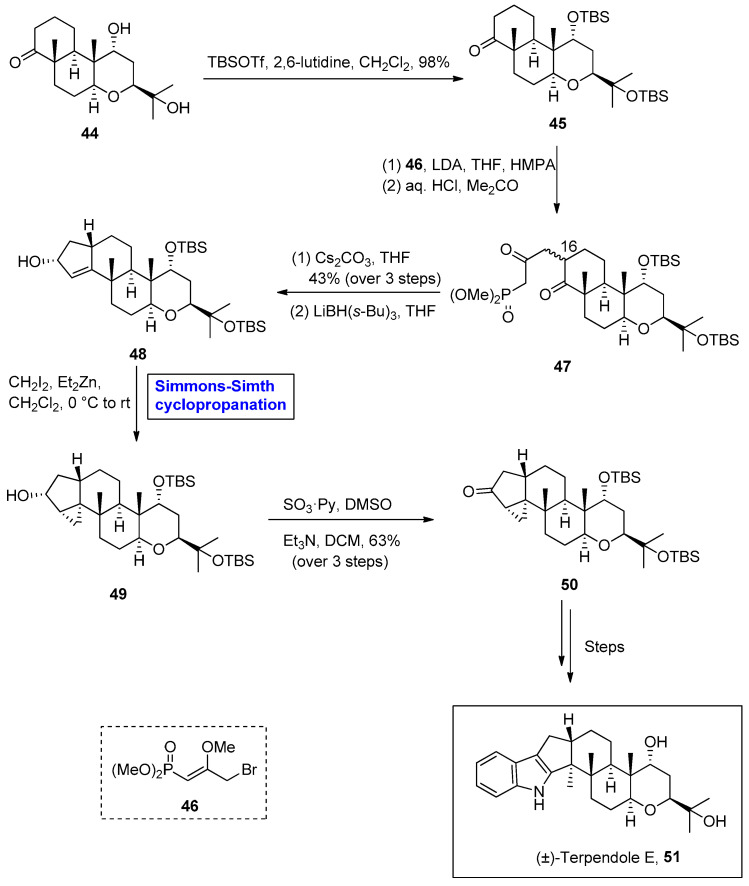
Synthesis of (±)-terpendole E **51**.

Terpendole E **51** belongs to the class of indole diterpenes and is isolated from fungus *Albophoma yamanashiensis*. It is the first natural mitotic kinesin Eg5 inhibitor [43], which acts as a weak ACAT (acyl-CoA: cholesterol acyltransferase) inhibitor [44]. Teranishi et al. in 2014 performed the first synthesis of the racemate of terpendole E over 13 steps, with a 13% yield [45]. In their methodology, compound **44** was silylated using TBSOTf to result in the desilylated product **45** (in an 89% yield over two steps). The reaction of compound **45** with reagent **46**, followed by hydrolysis at C16, produced compound **47**. In the next step, cyclization occurred, followed by reduction, to yield compound **48**. The Simmons–Smith cyclopropanation of allylic alcohol **48** in the presence of Et_2_Zn and CH_2_Cl_2_ resulted in the successful installation of a stereocenter at the C3 position of compound **49,** proceeding oxidation to yield intermediate **50** with a 64% yield. This intermediate was further modified in a few steps to yield the final product, (±)-terpendole E **51** (Scheme 7).

**Scheme 8:**
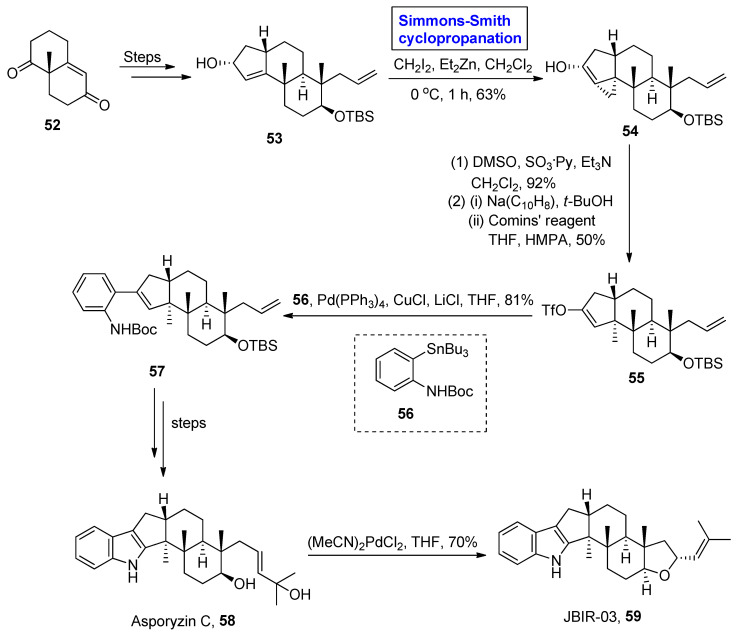
Synthesis of asporyzin C **58** and JBIR-03 **59**.

JBIR-03 **59** and asporyzin C **58** both belong to the class of indole diterpenes. These are isolated from *Aspergillus oryzae*. The molecular architecture of JBIR-03 contains a tetrahydrofuran ring adjacent to its hexacyclic ring. It exhibits antifungal, anti-MRSA and insecticidal activities. It does not show any cytotoxicity against fibrosarcoma cells of a human cell line (HT-1080). Asporyzin C specifically shows antibacterial activity against *E*. *coli* [46,47]. No synthetic strategy has previously been designed for the synthesis of these two attractive pharmacologically important compounds. In 2018, Murokawa et al. for the first time performed the synthesis of JBIR-03 **59** and asporyzin C **58** in 13 to 14 steps [48]. The intermediate in the synthesis of these compounds contains a cyclopropane ring. In their synthetic strategy, bicyclic keto alcohol **52** was used as the starting material and modified into compound **53** in a few steps. The installation of a cyclopropane ring by using the Simmons–Smith protocol was directed by a hydroxy group. For this purpose, compound **53** was treated with CH_2_I_2_, Et_2_Zn and CH_2_Cl_2_ as a solvent at 0 °C for 1 h to produce compound **54** with a 63% yield. For the installation of a methyl group at the C3 position, Parikh–Doering oxidation of compound **54** was performed, followed by the reductive cleavage of the cyclopropane ring by reacting it with sodium naphthalenide in THF as a solvent, furnishing compound **55**. The Stille coupling of compound **55** with stannane **56**, followed by a Pd-catalyzed indole ring formation, resulted in the synthesis of compound **57**. Asporyzin C **58** was formed by treating compound **57** over numerous steps. After that, the palladium-catalyzed ring closure of compound **58** yielded JBIR-03 **59** in a 70% yield (Scheme 8).

#### 2.2.2. Sesquiterpenes

**Scheme 9:**
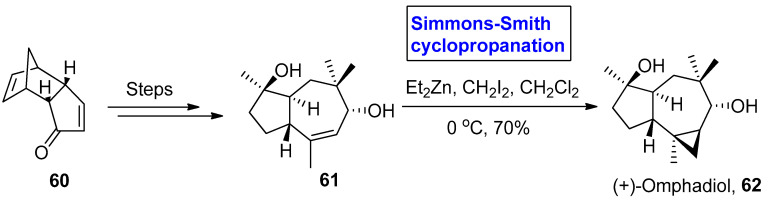
Total synthesis of (+)-omphadiol **62**.

**Scheme 10:**
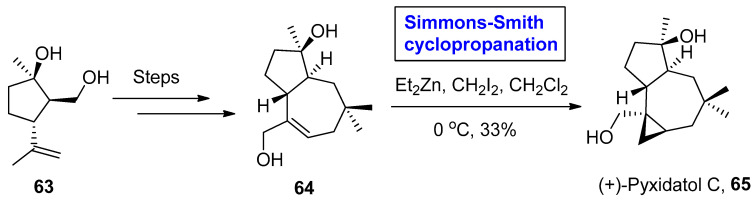
Total synthesis of (+)-pyxidatol C **65**.

(+)-Omphadiol **62** belongs to the class of sesquiterpenes, which possess antibacterial properties, and can be isolated from *Omphalotus illudosin*. (+)-Omphadiol **62** has a tricyclic structure, and it contains six adjacent stereogenic centers [49]. The total synthesis of (+)-omphadiol was for the first time performed by Romo and his colleagues in a 12-step sequence and an 18% yield [50]. Various other attempts at the synthesis of this heterocycle have also been reported. However, the introduction of six adjacent chiral centers has always remained a challenging task. In 2016, Parthasarathy et al. performed the synthesis of (+)-omphadiol starting from norbornene derivative **60,** which after a series of steps yielded intermediate **61** [51]. The stereoselective Simmons–Smith cyclopropanation of the C2−C4 double bond of intermediate **61** in the presence of Et_2_Zn and CH_2_Cl_2_ at 0 °C produced (+)-omphadiol **62** in a 70% overall yield (Scheme 9).

(+)-Pyxidatol C **65** is also a sesquiterpene. It was isolated from *Clavicorona pyxidate*, which is a mushroom. It is a widely used medicine for the treatment of dyspepsia, gastric pain and heat toxicity. It has four adjoining stereogenic centers [52,53]. Parthasarathy et al. performed the synthesis of (+)-pyxidatol C **65** starting from diol **63**, which (in a series of steps) was converted into intermediate **64** [51]. The Simmons–Smith cyclopropanation of allylic alcohol **64** produced (+)-pyxidatol C **65** in a 33% overall yield (Scheme 10).

**Scheme 11:**
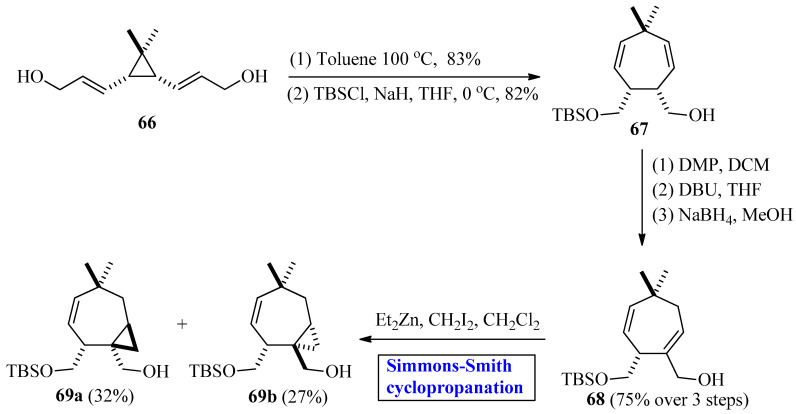
Synthesis of cycloheptadiene intermediate (**69a** and **69b**) toward the total synthesis of pyxidatol C.

Considering the pharmaceutical importance of pyxidatol C **65**, another group, Osler et al. in 2016, also disclosed a synthetic strategy for its synthesis [54]. In their methodology, *gem*-dimethyl-substituted divinyl cyclopropanes were used as starting materials. The Cope rearrangement of compound **66** produced a substituted cycloheptadiene **67**. The oxidation of diol **67**, followed by treatment with DBU and THF at 0 °C and subsequent reduction by using NaBH_4_, produced compound **68**. In the next step, the installation of a cyclopropane ring was achieved by using the Simmons–Smith protocol in the presence of diethyl zinc, dichloromethane and diiodomethane at 0 °C, successfully producing a diastereomeric mixture of compounds **69a** and **69b** with 32% and 27% respective yields. These compounds act as precursors for the total synthesis of pyxidatol C **65** (Scheme 11).

**Scheme 12:**
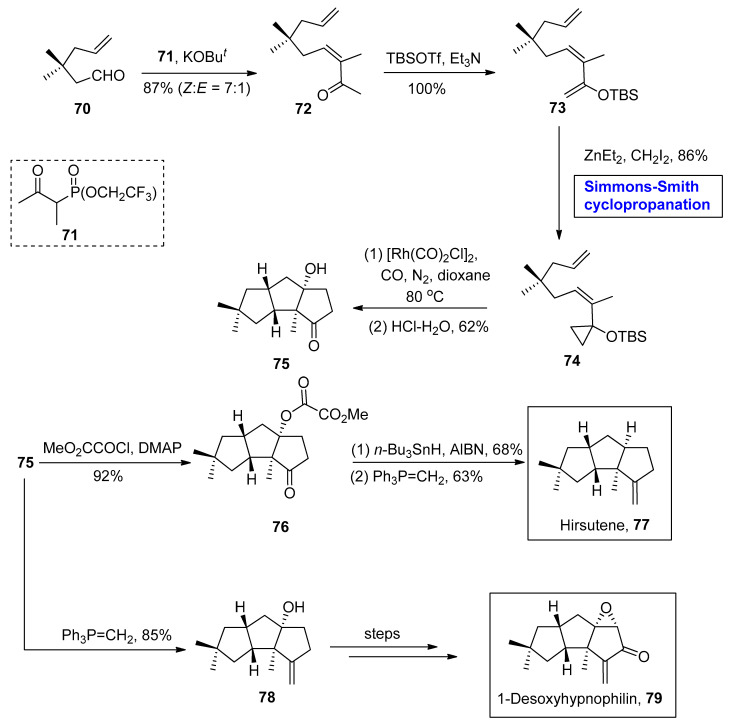
Synthesis of hirsutene **77** and 1-desoxyhypnophilin **79**.

Hirsutene **77** and 1-desoxyhypnophilin **79** are linear triquinanes, which belong to the class of sesquiterpenoids [55]. Their natural sources are plants, microbes and marine organisms. Their tricyclic skeletons exhibit numerous biological activities [56]. In 2007, Jiao et al. disclosed an efficient, concise and straightforward strategy for the diastereoselective synthesis of hirsutene **77** and 1-desoxyhypnophilin **79** in eight-step (11% overall yield) and nine-step (13% overall yield) sequences, respectively [57]. The key steps for the synthesis of intermediate **75** (with desired stereochemistry at two quaternary stereodefined centers) entailed HWE olefination, Simmons–Smith cyclopropanation and rhodium-catalyzed cycloaddition reaction. Their synthesis commenced with the Horner–Wadsworth–Emmons (HWE) reaction of dimethylhexenal **70** (as an easily available starting material) with phosphonate carbanion **71**, which afforded compound **72** in an 87% yield. Compound **72** was then silylated to produce compound **73**, which proceeded further toward chemoselective cyclopropanation in the presence of diethyl zinc and diiodomethane (Simmons–Smith protocol) to obtain compound **74** in an 86% yield. In the next step, Rh-catalyzed (5+2+1) cycloaddition of compound **74**, followed by aldol condensation, resulted in intermediate **75** in a 62% yield. For the synthesis of hirsutene **77**, intermediate **75** proceeded to acylation (in the presence of methyl oxalyl chloride and DMAP) to produce compound **76** in a 92% yield. Deoxygenation of compound **76** and a subsequent Wittig reaction completed the total synthesis of our desired compound **77** in a quantitative yield. In another route, a Wittig reaction of intermediate **76** afforded compound **78** in an 85% yield. The modification of compound **78** was performed in a number of steps to complete the total synthesis of 1-desoxyhypnophilin **79** (Scheme 12).

**Scheme 13:**
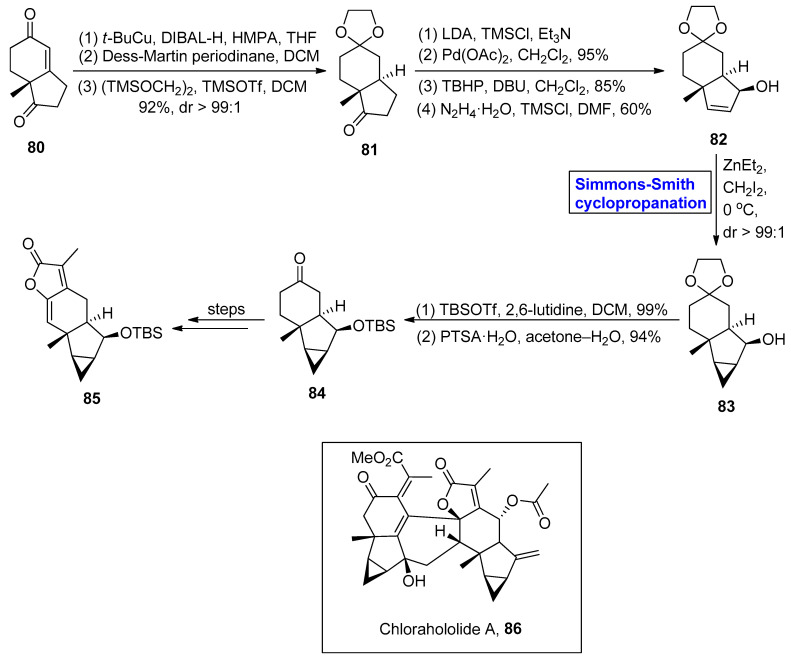
Synthesis of intermediate **85** toward the total synthesis of chlorahololide A **86**.

**Scheme 14:**
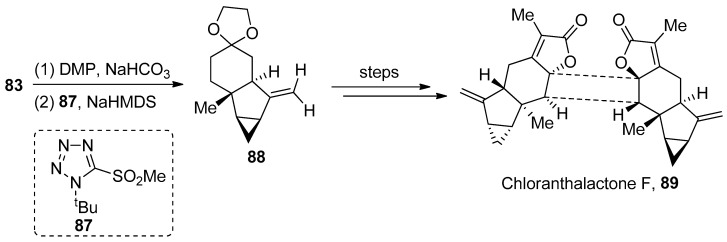
Synthesis of intermediate **88** toward the total synthesis of (+)-chloranthalactone F **89**.

Chlorahololide A **86** belongs to the class of sesquiterpenoid dimers. It was first isolated by Yue and his colleagues from *Chloranthus holostegius* in 2007 [58]. It is a rectifier potassium ion current inhibitor (IC_50_ = 10.9 μM) and is important for the treatment of many diseases [59]. In 2010, Qian and Zhao disclosed the synthesis of key intermediate **85** toward the synthesis of chlorahololide A **86** by employing Simmons–Smith cyclopropanation as a key step [60]. Their synthesis began with the reduction of Hajos Parrish ketone **80** (starting material), then treatment with Dess–Martin periodinane and subsequent protection via (TMSOCH_2_)_2_, resulting in compound **81** in a 92% yield. Saegusa oxidation of ketone **81**, followed by epoxidation and subsequent Wharton transposition, furnished compound **82** in a 60% yield. In the next step, the stereochemistry at the C-10 methyl group was carefully controlled by hydroxyl-induced Simmons–Smith cyclopropanation in the presence of diethyl zinc and diiodomethane to furnish compound **83** with excellent diastereoselectivity and successful installation of five stereocenters. Furthermore, desilylation in the presence of TBSOTf and removal of the glycol group transformed compound **83** into compound **85**, which over a few steps furnished our desired compound (Scheme 13).

(+)-Chloranthalactone F **89** belongs to the class of lindenane sesquiterpenoids (dimers). These are isolated from the chloranthus glaber plant [61]. Their structural framework comprises two cyclopropane rings, a cyclobutane ring, two double bonds present outside the ring and twelve stereogenic centers [62]. In 2012, Qian and Zhao revealed the enantioselective synthesis of (+)-chloranthalactone F **89** in 14 steps [63]. The main steps of their synthetic scheme involved Simmons–Smith cyclopropanation and chromium-trioxide-catalyzed oxidative lactonization followed by oxidative enol-lactonization. The cyclopropanated compound **83** (which was synthesized via employment of Simmons–Smith cyclopropanation, as shown in Scheme 13) was oxidized in the presence of Dess–Martin periodinane and NaHCO_3_, followed by methylation in the presence of reagent **87** to obtain compound **88**. After a few steps, successful synthesis of the final product **89** (with a 92% overall yield) was achieved (Scheme 14).

**Scheme 15:**
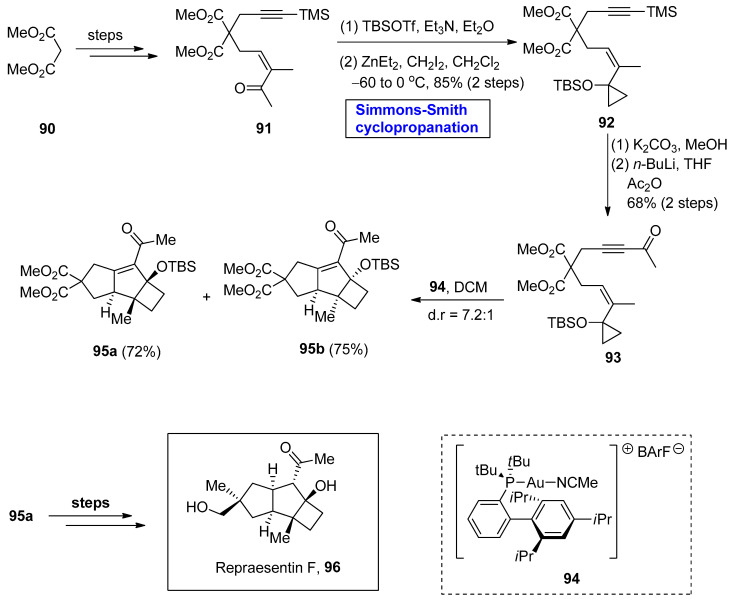
Total synthesis of repraesentin F **96**.

Repraesentin F **96** belongs to the class of sesquiterpenes. It was first isolated in 2006 from the fruiting bodies of an endemic fungus, *Lactarius repraesentaneus*, found in Japan [64]. This tricyclic scaffold plays an important role in regulating plant growth [65]. In 2018, Ferrer and Echavarren [66] proposed the first total synthesis of repraesentin F **96** over 16 steps, in a 2% overall yield, by employing Simmons–Smith cyclopropanation and gold-catalyzed cyclization as key steps. The synthesis commenced with dimethyl malonate **90** as a starting material, which was modified into 1,6-enyne **91** over a few steps. Compound **91** was then treated with TBSOTf and triethyl amine, followed by the addition of Simmons–Smith reagent, i.e., diethyl zinc, diiodomethane and dichloromethane, at a temperature below 0 °C, which furnished compound **92** with the successful incorporation of a cyclopropane ring. In the next step, deprotection in the presence of the base and subsequent acylation produced compound **93** (in a 68% yield), which proceeded further via gold-catalyzed cyclization by adding 5 mol% of catalyst **94** to furnish compounds **95a** and **95b** in 72% and 75% respective yields (with d.r = 7.2:1). The modification of compound **95a** over a few steps completed the tricyclic core synthesis of repraesentin F **96** (Scheme 15).

#### 2.2.3. Diterpenoids

**Scheme 16:**
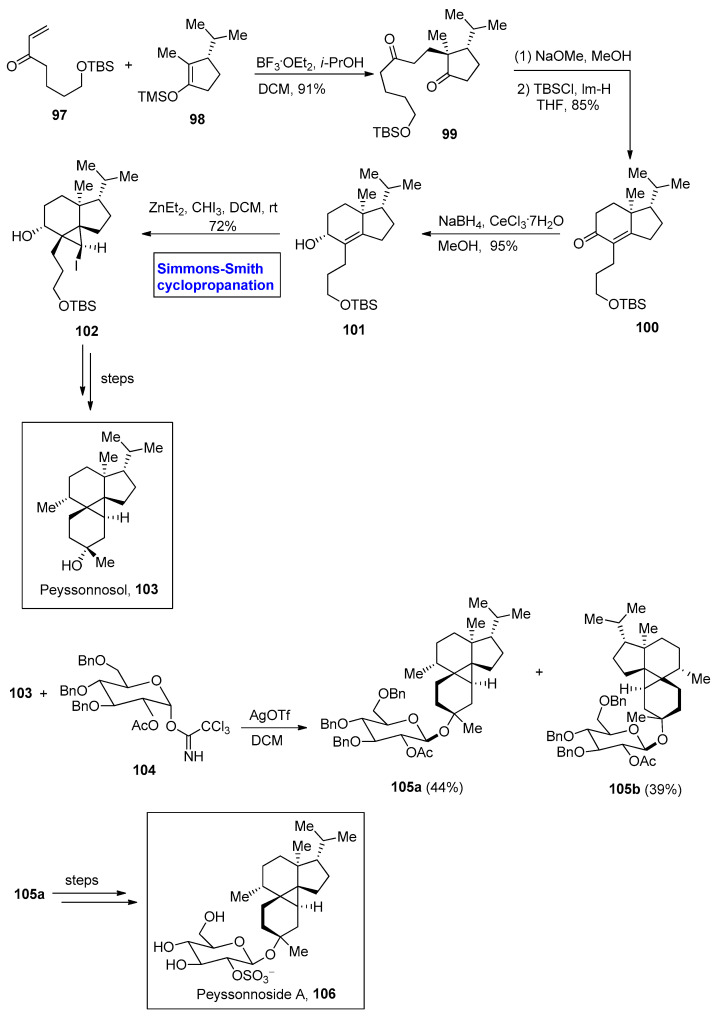
Synthesis of peyssonnosol **103** and peyssonnoside A **106**.

Peyssonnoside A **106** is a marine sulfated β-linked diterpenoid glucoside. It was first isolated from *Peyssonnelia* sp. of a red alga by Kubanek and coworkers in 2019 [67]. This sulfated diterpenoid glucoside has a tetracyclic structure that contains two hexacyclic rings, one pentacyclic ring and a sterically embedded (pentasubstituted) cyclopropane ring. It contains six out of seven adjoining stereogenic centers and three quaternary stereocenters. It is used for the treatment of liver diseases and shows potent biological activities against *Staphylococcus aureus* and *Plasmodium berghei* [68]. In 2019, Chesnokov and Gademann [69] performed the total synthesis of peyssonnoside A **106** for the first time in a very efficient, concise and diastereoselective fashion. It was achieved in 12 steps with a 21% overall yield from easily available starting materials (compounds **97** + **98**). In their methodology, compounds **97** and **98** were processed through a Mukaiyama-type Michael addition to produce compound **99**. In the next step, treatment of compound **99** with NaOMe and MeOH completed the construction of a six-membered ring via Robinson annulation followed by desilylation in the presence of TBSCl and imidazole, which resulted in the synthesis of bicyclic enone **100** in an 85% yield. Compound **100** was then reduced in the presence of NaOMe and MeOH to produce allylic alcohol **101** in a 95% yield. In order to construct a three-membered ring, compound **101** was cyclopropanated by using Simmons–Smith reagent, i.e., diethyl zinc in DCM as a solvent. The directing effect of alkoxide was carefully controlled by the addition of iodoform at room temperature, which yielded the single isomer **102** in a 72% yield. Compound **102** was modified in a few steps to furnish racemic peyssonnosol **103** (Scheme 16), which was then processed through β-selective Schmidt glucosylation with compound **104** to produce a diastereomeric mixture of **105a** and **105b**. The mixture of both these glucosides was separated, after which compound **105a** was processed further for modification over a few steps to complete the synthesis of our desired peyssonnoside A **106** (Scheme 16).

**Scheme 17:**
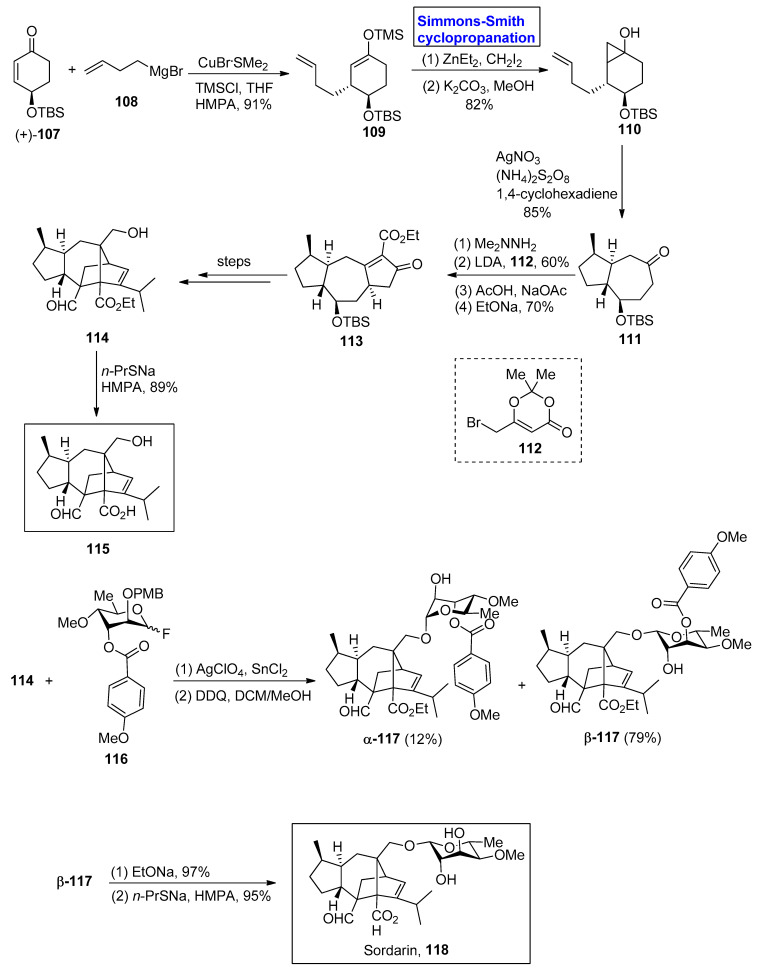
Total synthesis of sordaricin **115** and sordarin **118**.

Sordarin **118** was first isolated from *Sordaria araneosa*, a fungus, in 1971. It exhibits antifungal activities against *Candida albicans* [70]. The complex molecular architecture of sordarin **118** consists of a tetracyclic core, named sordaricin **115**. Sordaricin **115** belongs to the class of diterpenes. Its structure is based on a norbornene framework with three adjacent stereogenic centers [71]. Considering the unique structural features and interesting biological activities of sordarin **118**, in 2006, Chiba et al. planned and reported its total synthesis [72]. To perform this task, optically active cyclohexanone (+)-**107** was allowed to react with 3-butenylmagnesium bromide **108** under given conditions to produce compound **109**. In the next step, Simmons–Smith cyclopropanation of compound **109** (in the presence of diethyl zinc and diiodomethane) and subsequent treatment with the base for desilylation resulted in cyclopropanol **110** in an 82% yield. Compound **110** was processed further for a cyclopropanol ring opening and oxidative radical cyclization in the presence of silver nitrate and 1,4 cyclohexadiene to furnish compound **111** in an 85% yield. The synthesis of tricyclic intermediate **113** (in a 70% yield) involved reaction of ketone **111** with *N*,*N*-dimethylhydrazine to produce *N*,*N*-dimethylhydrazone (intermediate), processed by reaction with compound **112** followed by acetonide deprotection and subsequent condensation in the presence of sodium ethanoate. Over a few steps, intermediate **113** was transformed into compound **114**, which upon deethylation resulted in sordaricin **115**. For the synthesis of sordarin **118**, Mukaiyama’s coupling of sordaricin ethyl ester **114** with glycosyl fluoride **116** and subsequent treatment with DDQ was performed, which furnished a mixture of **α-117** and **β-117** as a major product in 12% and 79% yields, respectively, with a good diastereoselective ratio (dr = 6.5:1). In the next step, **β-117** was subjected to deprotection in the presence of EtONa, and subsequent deethylation completed the synthesis of sordarin **118** (Scheme 17).

**Scheme 18:**
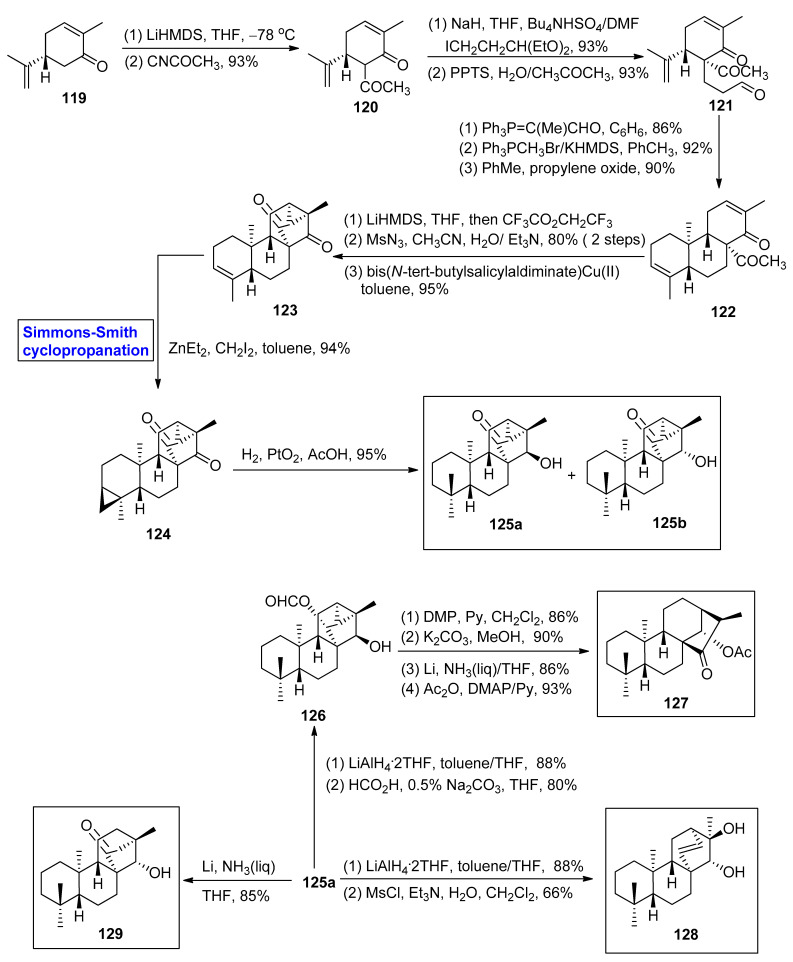
Synthesis of trachylobane (**125a** and **125b**), kaurane **127**, atisane **128** and beyerane framework **129**.

Trachylobanes (**125a** and **125b**), kaurane **127**, atisane **128** and beyerane **129** belong to the class of polycyclic diterpenes [73,74]. These tetracyclic diterpenes exhibit promising biological activities. In 2006, Abad et al. revealed a synthetic route toward the synthesis of these fascinating heterocycles [75]. Their methodology was based on the synthesis of common intermediate **124** by employing Simmons–Smith cyclopropanation as a key step. In the first step, the (*R*)-carvone **119** (as a starting material) was reacted with acetaldehyde, which then underwent Swern oxidation to produce β-diketone **120** (in a 93% yield). Compound **120** was processed by treatment with NaH and Bu_4_NHSO_4_ in DMF, followed by alkylation and subsequent acidic hydrolysis in the presence of PPTS, resulting in compound **121**. In the next step, a Wittig reaction of compound **121** followed by Wittig methylation and a subsequent Diels Alder reaction furnished compound **122** in a 95% yield. Compound **122** was then transformed into compound **123** under the given conditions. In the next step, the well-suited Simmons–Smith cyclopropanation protocol was applied by adding diethyl zinc and diiodomethane in the presence of toluene, furnishing the desired intermediate **124**. For the synthesis of trachylobanes (**125a** and **125b**), regioselective reduction of intermediate **124** was performed by using hydrogen gas in the presence of platinum as a catalyst and AcOH as a solvent, thus resulting in a mixture of **125a** and **125b** in a 95% (combined) yield. In the following step, compound **125a** was modified into compound **126** under the given conditions. Compound **126** was processed by oxidation in the presence of Dess–Martin periodinane, followed by hydrolysis and treatment with liquid ammonia in the presence of THF, and subsequent acetylation furnished kaurane **127** in an excellent yield. For the synthesis of atisane **128**, compound **125a** was reduced in the presence of lithium aluminum hydride and then subsequently mesylated, while in another route, **125a** was treated with liquid ammonia to furnish the desired beyerane **129** in an 85% yield (Scheme 18).

#### 2.2.4. Triterpenoids

**Scheme 19:**
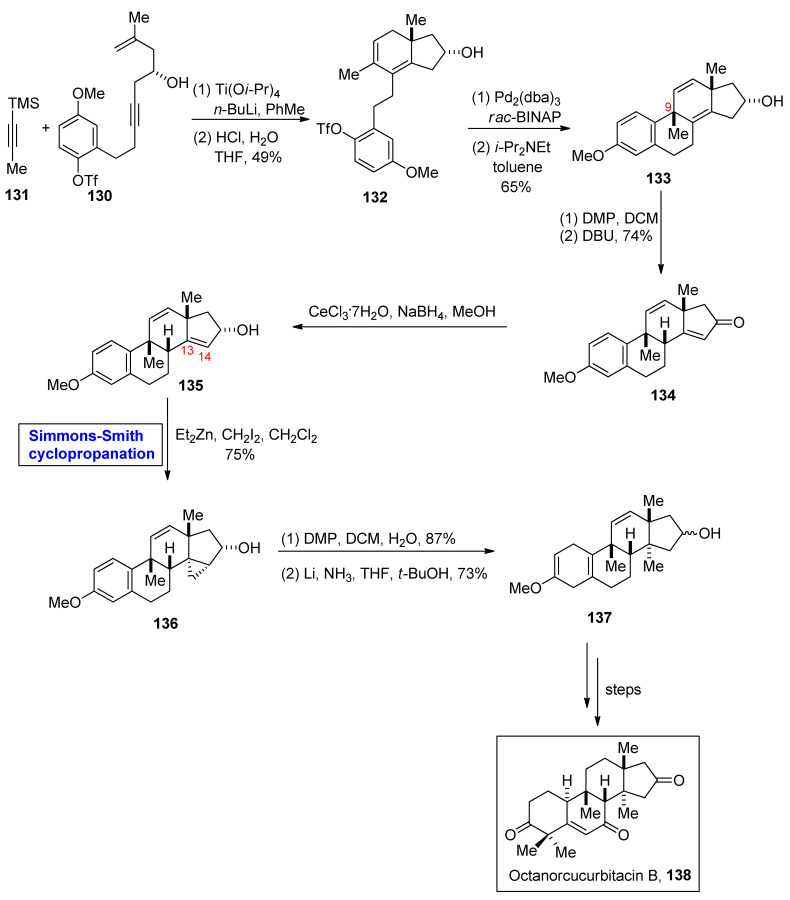
Synthesis of octanorcucurbitacin B **138**.

Octanorcucurbitacin B **138** belongs to the class of cucurbitane triterpenoids. These are isolated from the plants of *Momordica charantia* [76]. The cucurbitane class of triterpenoids are important for exhibiting anti-inflammatory, antitumor and anti-HIV activities. These show structural similarity with euphanes and lanostanes as far as possessing tetracyclic skeletons with three stereocenters. In the case of cucurbitanes, these three quaternary centers are present at the C9, C13 and C14 positions [77,78]. Previous approaches toward the synthesis of octanocucurbitacin were based on cationic-rearrangement-mediated derivatization of lanostanes. However, lanostanes were not readily available starting materials. In 2022, Bucknam et al. accomplished the stereoselective synthesis of octanorcucurbitacin B **138** from readily available chiral enyne **130** in 12 steps with a 0.8% overall yield [79]. In their synthetic strategy, compound **130** was allowed to react with TMS-propyne **131**, and then protodesilylation produced compound **132**. In order to generate a stereocenter at the C9 position, compound **132** underwent a Heck reaction to produce polyunsaturated tetracycle **133**. In the next three steps, oxidation (by using Dess–Martin periodinane), isomerization (in the presence of DBU) and subsequent reduction (in the presence of NaBH_4_) were performed to furnish compound **135** with a stereocenter at C8. The development of a C14 stereocenter was somehow difficult; hence, the Simmons–Smith cyclopropanation strategy was employed to achieve this task. Compound **135** was reacted with diethylzinc and diiodomethane to produce compound **136** as a single regioisomer in a 75% yield. In the next step, oxidation of compound **136** and subsequent reduction (in the presence of lithium in ammonia) successfully generated compound **137** with a chiral center at C14. In the next few steps, a series of oxidation and reduction reactions were performed to furnish our desired natural product **138** in a good yield (Scheme 19).

### 2.3. Synthesis of Amino-Acid-Based Natural Products

**Scheme 20:**
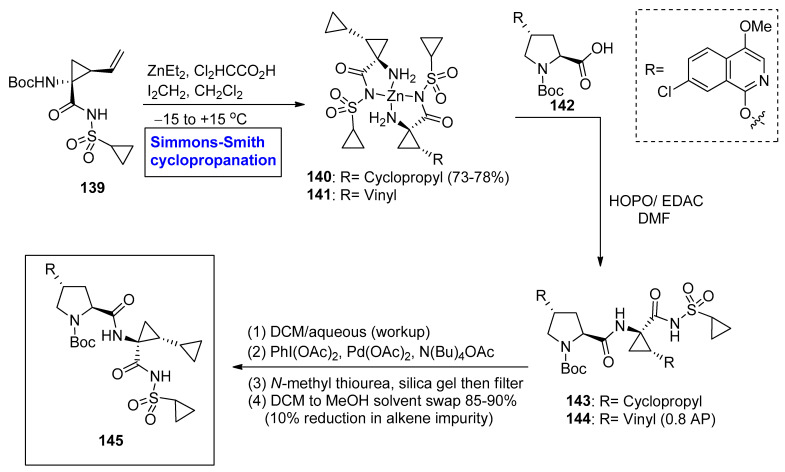
Synthesis of tricyclopropylamino acid derivative as an active pharmaceutical ingredient (API) **145**.

The tricyclopropylamino acid derivative is an active pharmaceutical ingredient (API) **145** consisting of proline and tricyclopropylamino acid. It is expected to be used in the treatment of hepatitis C [80]. Previously, tricyclopropylamino acid was synthesized by palladium-catalyzed cyclopropanation reactions in the presence of diazomethane. However, using diazomethane on a kilogram scale was not a good choice because of its hazardous nature. Another common problem faced by many researchers during API **145** synthesis was incomplete cyclopropanation that resulted in alkene impurity [81]. Young et al. investigated many strategies for large-scale synthesis of compound **145** in high purity [82]. In 2016, they designed a useful strategy by employing the use of well-suited Simmons–Smith cyclopropanation as a key step with an additional aminoacetoxylation process. In their synthetic route, compound **139** underwent Simmons–Smith cyclopropanation by using 3.39 equivalents of diethyl zinc and 3.20 equivalents of diiodomethane and dichloroacetic acid to produce compound **140** (or **141**) at −15 to 15 °C with a 70–80% yield. Compound **140**/**141** was then reacted with a Boc-protected proline derivative in the given conditions to obtain compound **143/144** and processed by treatment with PhI(OAc)_2_, Pd(OAc)_2_ and N(Bu)_4_OAc, and the subsequent addition of *N*-methyl thiourea resulted in the desired compound API **145** in an 85–90% yield. This amino acid derivative caused a reduction in alkene impurity from 20 to 0.12 AP (Scheme 20).

**Scheme 21:**
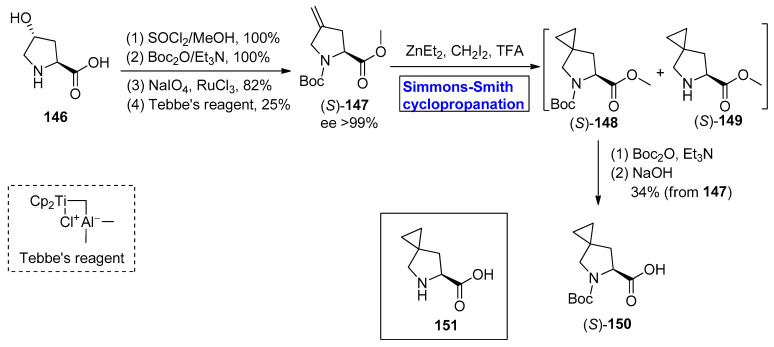
Synthesis of Boc-protected 5-azaspiro[2.4]heptane-6-carboxylic acid (*S*)-**150**.

Cyclopropane-ring-based amino acids are important in medicinal chemistry because of their wide biological activities. (6*S*)-5-azaspio[2.4]heptane-6-carboxylic acid **151** is a cyclopropane containing _L_-proline [83,84]. In 2012, Tymtsunik et al. disclosed the enantiomeric synthesis of Boc-protected 5-azaspiro[2.4]heptane-6-carboxylic acid **150** in six steps by using (2*S*,4*R*)-4-hydroxyproline **146** as a starting material and by employing Simmons–Smith cyclopropanation as a key step [85]. The overall yield was 5% with good control of chirality. In their methodology, compound **146**, under the given conditions (a Wittig reaction and treatment with Tebbe’s reagent), was modified into compound (*S*)-**147** in a 25% yield with ee > 98%. In the next step, the Simmons–Smith protocol was employed, for which compound **147** was treated with ZnEt_2,_ CH_2_I_2_ and trifluoroacetic acid as a solvent, furnishing a mixture of compound (*S*)-**148** (in a 34% yield) and compound (*S*)-**149**. In the last step, Boc protection and subsequent hydrolysis (in the presence of NaOH) of compound **148** completed the synthesis of compound (*S*)-**150** as a single enantiomer (Scheme 21).

**Scheme 22:**
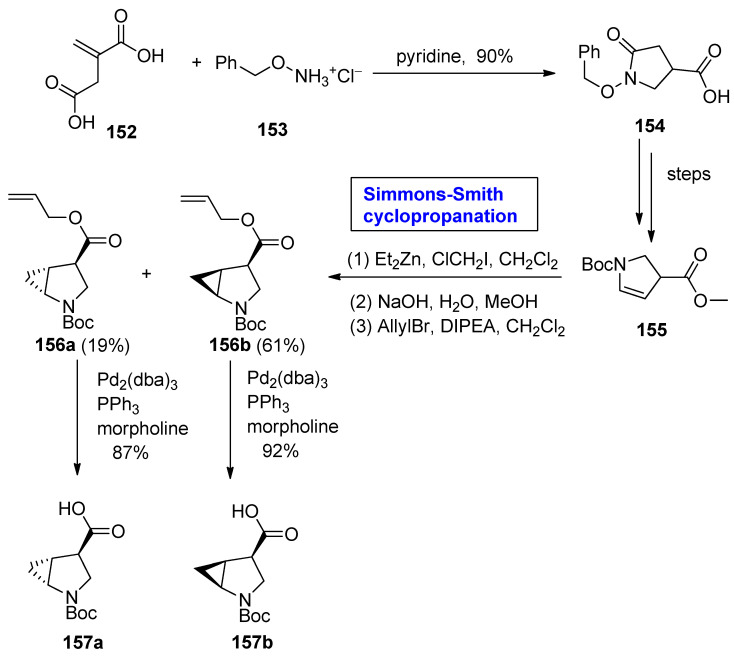
Synthesis of Boc-protected 4,5-methano-β-proline (**157a** and **157b**).

Boc-protected 4,5-methano-β-proline (**157a** & **157b**) is another analogue of β-amino acid. It is used in the synthesis of antidiabetic drugs, i.e., saxagliptin [86,87]. In 2014, Tymtsunik et al. [88] performed the diastereomeric synthesis of Boc-protected 4,5-methano-β-proline (**157a** & **157b**) by employing the Furukawa variation of Simmons–Smith cyclopropanation as a key step. Itaconic acid **152** was used as a starting material, and the overall yields of resulting cis and trans isomers were 11% and 38%, respectively (total of 49%). In the first step, itaconic acid **152** was allowed to react with *O*-benzylhydroxylamine **153** (acting as nucleophile) to produce compound **154**. Over a few steps, compound **155** was allowed to react with 2.5 moles of ZnEt_2_ and 2.55 moles of ClCH_2_I in CH_2_Cl_2_ acting as a solvent, followed by hydrolysis and reaction with allyl bromide to produce a diastereoisomeric mixture of compounds **156a** and **516b** (after chromatographic separation). In the last step, deprotection of compounds **156a** and **156b** in the presence of Pd_2_(dba)_3_ and PPh_3_ furnished Boc-protected proline derivatives **157a** (87%) and **157b** (92%), respectively (Scheme 22).

**Scheme 23:**
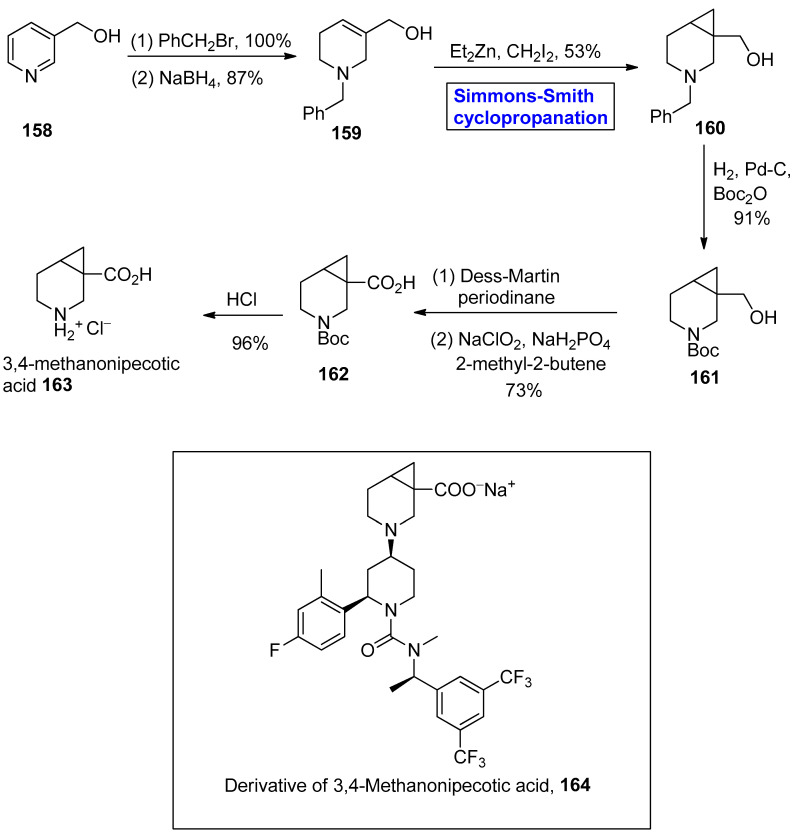
Synthesis of racemic 3,4-methanonipecotic acid **163**.

3,4-methanonipecotic acid **163** is a cyclopropyl ring containing non-proteinogenic β-amino acid [89]. This compound is rarely present in higher organisms and is usually isolated from plants and bacterial sources. β-amino acids, after incorporation into peptides, show interesting conformational behavior, which prompted researchers to design the various structural analogues of these amino acids. Compound **164** (a derivative of 3,4-methanonipecotic acid **163**) is an antagonist of the NK1 receptor; thus, it exhibits potent biological activities [90]. In 2015, Tymtsunik et al. developed a novel approach for the synthesis of racemic 3,4-methanonipecotic acid **163** by using 3-pyridinylmethanol **158** as a starting material [91]. The synthesis was performed via an eight-step sequence (in a 38% overall yield) by employing Simmons–Smith cyclopropanation as a key step. The treatment of alcohol **158** with Grignard reagent and subsequent reduction produced alcohol **159**. In the next step, compound **159** was processed by treatment with 4 moles of ZnEt_2_ and 4 moles of CH_2_I_2_ to yield compound **160** (with a successfully installed cyclopropane ring) in a 53% yield. In the next step, compound **160** was converted into compound **162** (in a 73% yield) over three steps involving reduction, followed by treatment with Dess–Martin periodinane and subsequent Pinnick oxidation (in the presence of 2-methyl-2-butene, NaClO_2_ and NaH_2_PO_4_). In the final step, deprotection of compound **162** via HCl furnished 3,4-methanonipecotic acid **163** in a 98% yield (Scheme 23).

**Scheme 24:**
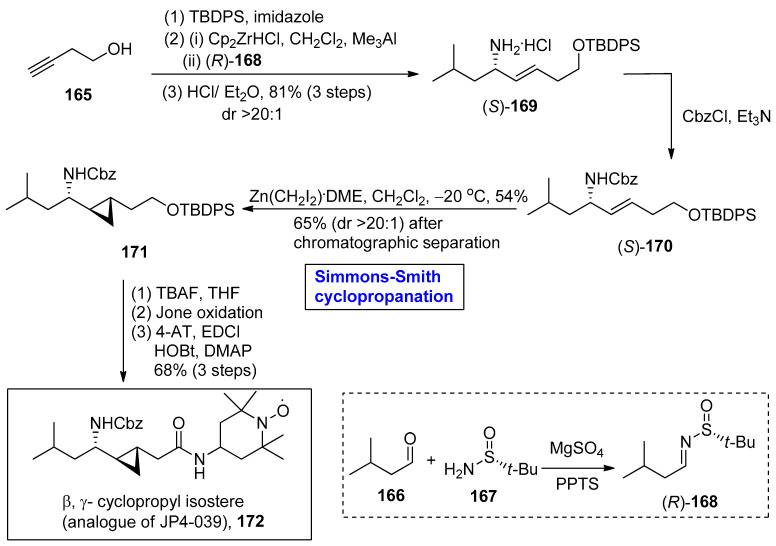
Synthesis of JP4-039 analogue **172**.

JP4-039 is an isostere dipeptide comprised of leucine and glycine residue [92]. Its structure possesses peptidomimetic properties and has the ability to interact with mitochondria and operate as a bioprotective and anti-oxidant agent [93]. In 2011, Frantz et al. designed an efficient and easily scaled route toward the synthesis of β, γ-cyclopropylamine isosteres **172** (analogue of JP4-039) [94]. In their synthetic route, compound (*S*)-**169** was obtained by hydrozirconation of alkyne **165** and simultaneous transmetalation, followed by reaction with chiral imine (*R*)-**168** (from isovaleraldehyde). In the next step, Cbz protection of allylic sulfinyl amine was performed to produce (*S*)-**170**. For cyclopropanation, compound (*S*)-**170** was treated with diethyl zinc and dichloromethane (Simmons–Smith reagent) at −20 °C and then with slight heating up to room temperature, followed by chromatographic separation, resulting in compound **171** in a 65% yield with dr >20:1. In the following step, compound **171** was treated with TBAF, followed by Jones oxidation and subsequent 4-AT coupling, furnishing our desired JP4-039 analogue **172** (over three steps) in a 68% yield (Scheme 24).

**Scheme 25:**
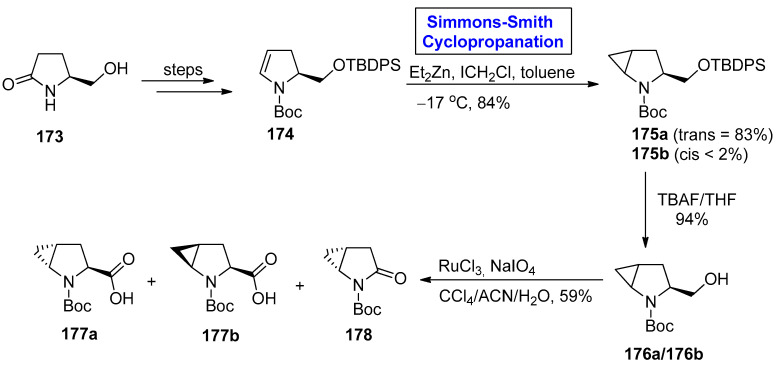
Synthesis of trans-methanoproline **177a**.

Methanoprolines belong to the class of amino acids; they have great medicinal importance and are expected to show anti-HCV activities [95,96]. In 2013, Wang et al. devised a new and efficient synthetic route for the synthesis of trans-methanoproline **177a** in high stereoselectivity [97]. Their synthesis commenced with readily available starting material **173**, which was modified into compound **174** in a few steps. In order to introduce a cyclopropane ring, a well-suited Simmons–Smith cyclopropanation was performed by treating compound **174** with Et_2_Zn and ICH_2_Cl in toulene at −17 °C to furnish a mixture of compounds **175a** and **175b** in an 84% yield. In the next step, silyl group deprotection of compounds **175a** and **175b**, followed by oxidation in the presence of sodium periodate and ruthenium chloride, furnished compounds **177a**, **177b** and **178** in a 1.0:0.04:0.18 mol ratio, respectively. After that, recrystallization was performed in order to achieve the desired compound **177a** in high stereoselectivity (Scheme 25).

### 2.4. Synthesis of Nucleosides

**Scheme 26:**
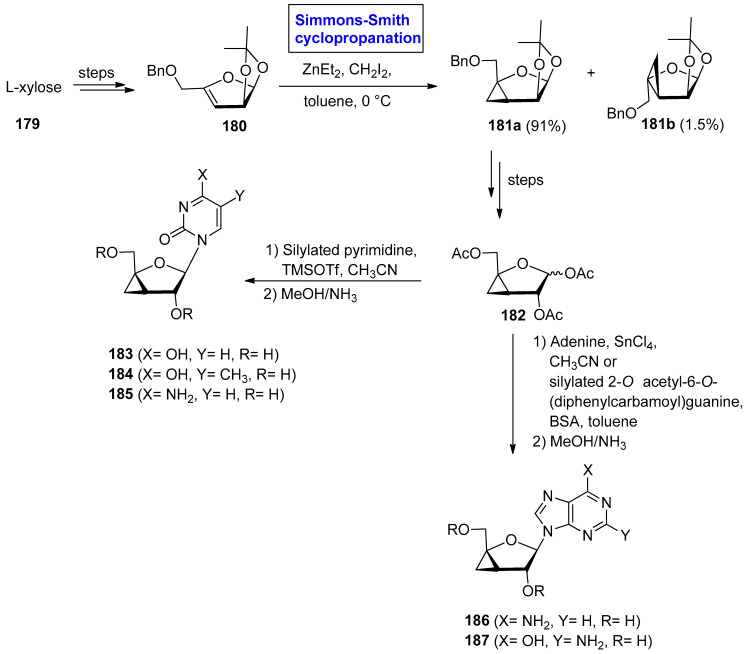
Synthesis of five 2-oxabicyclo[3.1.0]hexane-based nucleoside analogues.

2-oxabicyclo[3.1.0]hexane is a basic heterocyclic scaffold for the synthesis of various nucleoside analogues, as these show potent biological activities. The synthesis of these nucleosides involve glycosylation reactions between sugars bearing 2-oxabicyclo[3.1.0]hexane and nitrogenous bases. Conformational studies of these nucleosides and sugar-puckering phenomena are important, as these effect the metabolic behavior and interaction of these nucleosides with various polymerases [98]. Keeping in view the biological importance of nucleosides, Gangeron et al. in 2005 performed the synthesis of five 2-oxabicyclo[3.1.0]hexane-based natural nucleic acid bases [99]. Their synthesis involved glycosylation reactions between sugars bearing 2-oxabicyclo[3.1.0]hexane and nitrogenous bases starting from L-xylose **179**. Compound **180** was obtained after the modification of L-xylose. Then, the Simmons–Smith cyclopropanation of compound **180** resulted in a mixture of major product **181a** (91% yield) and minor product **181b** (1.5% yield). Compound **181a** after modification over a few steps produced compound **182**. The glycosylation reaction of compound **182** in the presence of pyrimidine at 0 °C followed by deprotection in the presence of MeOH and NH_3_ produced nucleosides **183**, **184** and **185**. In another route, glycosylation reaction of compound **182** in the presence of adenine and tin chloride followed by deprotection produced nucleoside **186**, while guanosine nucleoside **187** was prepared by condensation of **182** with 2-N-acetyl-6-*O*-(diphenylcarbamoyl)guanine in the presence of toluene and subsequent deprotection in the presence of MeOH and NH_3_. Conformational studies of these nucleosides confirmed their restriction toward ^o^T_1_ conformation (Scheme 26).

**Scheme 27:**
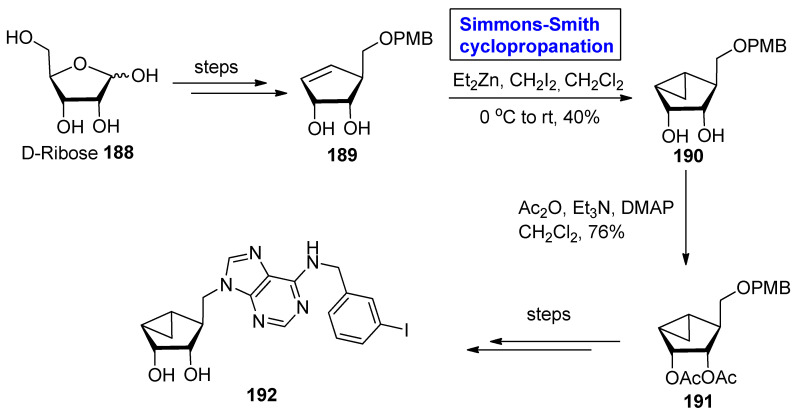
Synthesis of homologated (*N*)-methanocarba nucleoside **192**.

Methanocarba nucleosides contain bicyclo[3.1.0]hexane carbasugar and are able to mimic furanose ring puckering and are effective PPAR dual modulators [100]. The role of peroxisome proliferator-activated receptor (PPAR) dual modulators have previously been reported. These modulators can be used in the treatment of hypoadiponectinemia (a metabolic disease) and cancer [101,102]. These PPARδ antagonists and PPARγ partial agonists work by interacting with polymerases and adenosine receptors and inactivating them. Considering the importance of PPAR modulators, various attempts have been made for the synthesis of methanocarba nucleosides, but previous approaches for the synthesis of these nucleosides faced the problem of low yield. However, Hyuan et al. in 2021 performed the stereoselective synthesis of homologated (*S*)- and (*N*)-methanocarba nucleosides on a bicyclo[3.1.0]hexane template and observed the conformational behavior of these analogues for binding with PPAR [103]. The key step in the synthesis of (*N*) conformer involved Simmons−Smith cyclopropanation and a Mitsunobu reaction [104]. In their synthetic route, D-Ribose **188** was used as the starting material, which after few modifications produced compound **189**. The substrate-controlled Simmons–Smith cyclopropanation of diol **189** in the presence of diethyl zinc, diiodomethane and dichloromethane at 0 °C to rt produced compound **190** with a 40% yield. Compound **190** was then acetylated by treating with Ac_2_O, Et_3_N and DMAP in the presence of dichloromethane at 0 °C to 23 °C, yielding compound **191** in a 76% yield. After a few steps, the synthesis of homologated (*N*)-methanocarba nucleosides **192** was completed with 58% overall yield, respectively (Scheme 27).

**Scheme 28:**
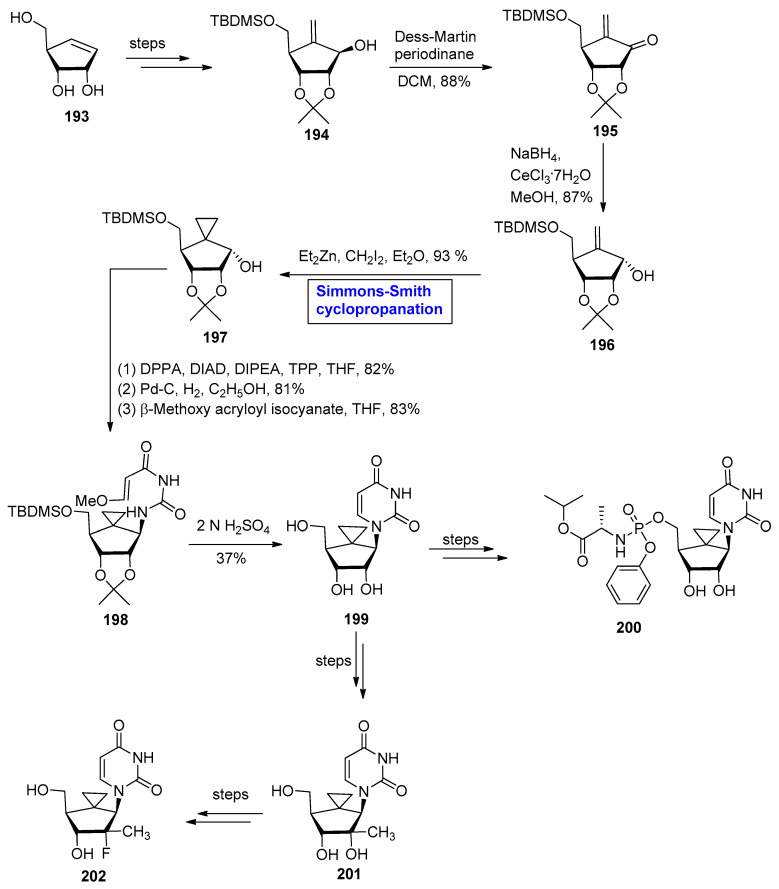
Synthesis of 1-(4*R*,5*S*,6*R*,7*R*)-5,6-dihydroxy-7-(hydroxymethyl)-.

20-deoxy-20-fluoro-20-C-methyl spiro cyclopentyl carbocyclic uridine belongs to the class of carbocyclic nucleosides, which are well known for their anticancer properties [105]. These nucleosides are generated by substituting oxygen in a furanose ring with carbon, and subsequent condensation with a base results in more stable nucleosides [106]. In 2020, Singh and Chu performed the synthesis of 1-(4 *R*,5*S*,6*R*,7*R*)-5,6-dihydroxy-7-(hydroxymethyl)-spiro[2.4]heptan-4-yl)pyrimidine-2,4(1H,3H)-dione **199** and its analogues and evaluated their anti-HCV activity [107]. Triol **193** (as a starting material) was modified into *β*-allylic alcohol **194** over a few steps. The oxidation of *β*-allylic alcohol **194** in the presence of Dess–Martin periodinane and dichloromethane produced *α*-allylic alcohol **195** (in an 88% yield), which upon subsequent reduction in the presence of sodium borohydride and cesium chloride produced compound **196** in an 87% yield. Compound **196** was cyclopropanated under the Simmons–Smith conditions, i.e., diethyl zinc, diiodomethane and diethyl ether, to furnish compound **197** in a 93% yield. Compound **197** was treated with Mitsunobu conditions [104], i.e., DPPA, DIAD and TPP in THF, to produce *β*-azide, which on subsequent reduction and then treatment with *β*-methoxy acryloyl isocyanate produced compound **198** in an 81% yield. The cyclization of compound **198** was performed in the presence of 2 N sulfuric acid to produce uridine analogue **199** successfully in a 37% yield. The phosphoramidate-containing derivative **200** and analogue **201** were synthesized from intermediate **199**. Compound **201** was further modified into analogue **202** (Scheme 28).

**Scheme 29:**
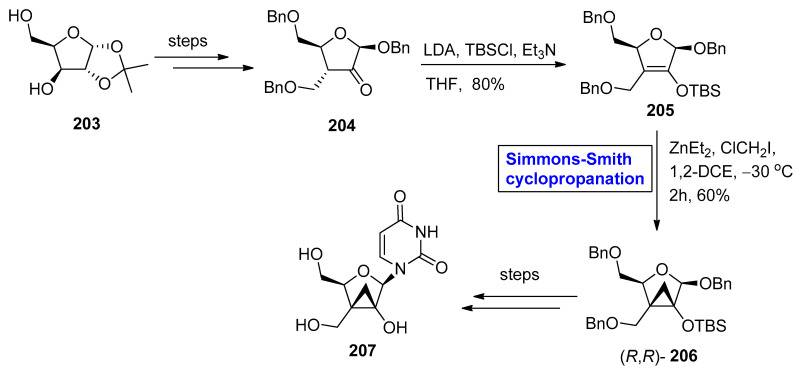
Synthesis of 3′-deoxy3′-C-hydroxymethyl-2′,3′-methylene-uridine **207**.

Spiro[2.4]heptan-4-yl)pyrimidine-2,4(1H,3H)-dione and analogues (**200–202**), 2′-C-methyl-uridine and 3′-C-hydroxymethyl-uridine, have been previously synthesized and evaluated for anti-HCV NS5B polymerase activity. A few works on the synthesis of 2′,3′-cyclopropane-bearing uridine have been reported in the literature [108,109]. The synthesis and stereochemical confirmation of 2′,3′-cyclopropane nucleoside, i.e., 3′-deoxy-3′-C-hydroxymethyl-2′,3′-methylene-uridine **207,** as an anti-HCV agent was reported by Komsta et al. in 2014 [110]. The synthesis was performed in 16 steps by using a readily available starting material, i.e., D-xylose derivative **203**. This acetonide-protected derivative **203**, after modifications in a few steps, produced protected 3-hydroxymethyl 2-ketofuranoside **204**. In the next step, the carbonyl group of derivative **204** was subjected to silyl protection in the presence of LDA, TBSCl, Et_3_N and THF at −30 °C to rt to produce benzyl-protected silyl enol ether in an 80% yield. Furthermore, Simmons–Smith cyclopropanation was performed according to the previously devised method of Gerber and Vogel, in which compound **205** was treated with diethyl zinc, ClCH_2_I and DCE to furnish compound **206** in a 60% yield and dr = 8:1. The modification of compound **206** over a few steps completed the synthesis of uridine **207** (Scheme 29).

**Scheme 30:**
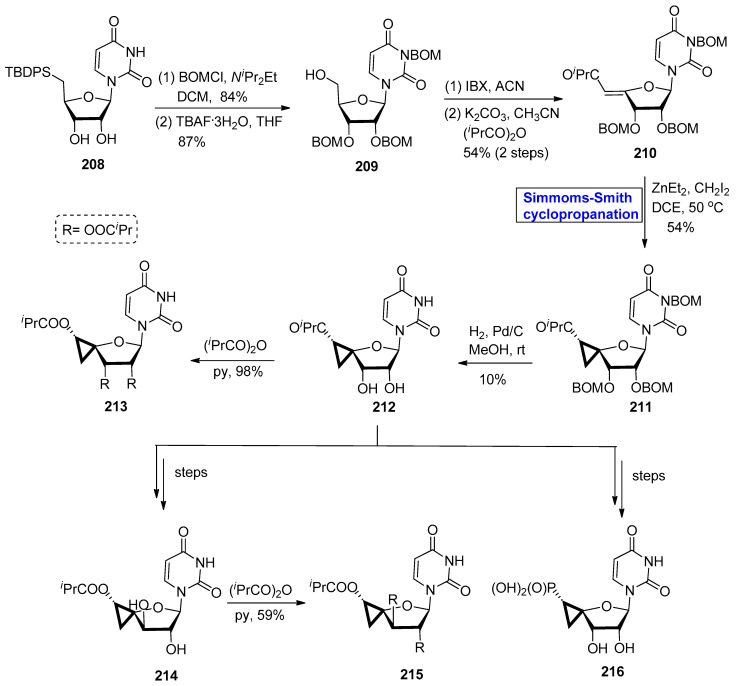
Synthesis of 4′/5′-spirocyclopropanated uridine and D-xylouridine derivatives (**213**–**216**).

There has been a great deal of interest in the synthesis of nucleoside derivatives and their use as antiviral agents against a broad range of viruses such as influenza, HIV-1, CMV, hepatitis C virus and human respiratory syncytial virus (HRSV). HRSV is a common cause of disease in both children and adults and in persons with weak immunity [111]. Keeping in view the role of nucleosides and the synthesis of their derivatives, 4′/5′-methylene spirocyclopropanated uridine has also been synthesized, but these synthetic procedures produced low yield [112]. In 2019, Kollmann et al. successfully synthesized 4′/5′-spirocyclopropanated uridine derivatives with a 5′ hydroxy substitution pattern using Furukawa-modified Simmons–Smith cyclopropanation as a key step [113]. In their methodology, *O*-silylated nucleoside **208** (as a starting material) was treated with six equivalents of BOMCl and five equivalents of N*^i^*Pr_2_Et at about 0 °C to room temperature, and subsequent treatment with TBAF produced compound **209** over two steps with an 87% yield. Oxidation of compound **209** by using IBX followed by enolization with K_2_CO_3_ produced compound **210**. After this, compound **210** underwent Simmons–Smith cyclopropanation in the presence of diethyl zinc, diiodomethane and DCE at 50 °C to produce compound **211** (in a 54% yield). In the following step, compound **211** was reduced by using Pd/C and methanol, which successfully yielded intermediate **212**. This spirocyclopropanated derivative **212** acted as a precursor for the synthesis of many other derivatives such as spirocyclopropanated uridine monophosphate (cpUMP) **216**, spirocyclopropanated D-xylouridine **214**, _D_-xylo nucleoside **215** and D-ribo nucleoside **213**. Compound **215** was synthesized by the esterification of compound **214** in the presence of isobutyric anhydride and pyridine (Scheme 30). All the synthesized derivatives, **212**, **213**, **214**, **215** and **216**, were evaluated for anti-HRSV activity. Among these synthesized compounds, two derivatives showed moderate anti-HRSV activity (Scheme 30).

**Scheme 31:**
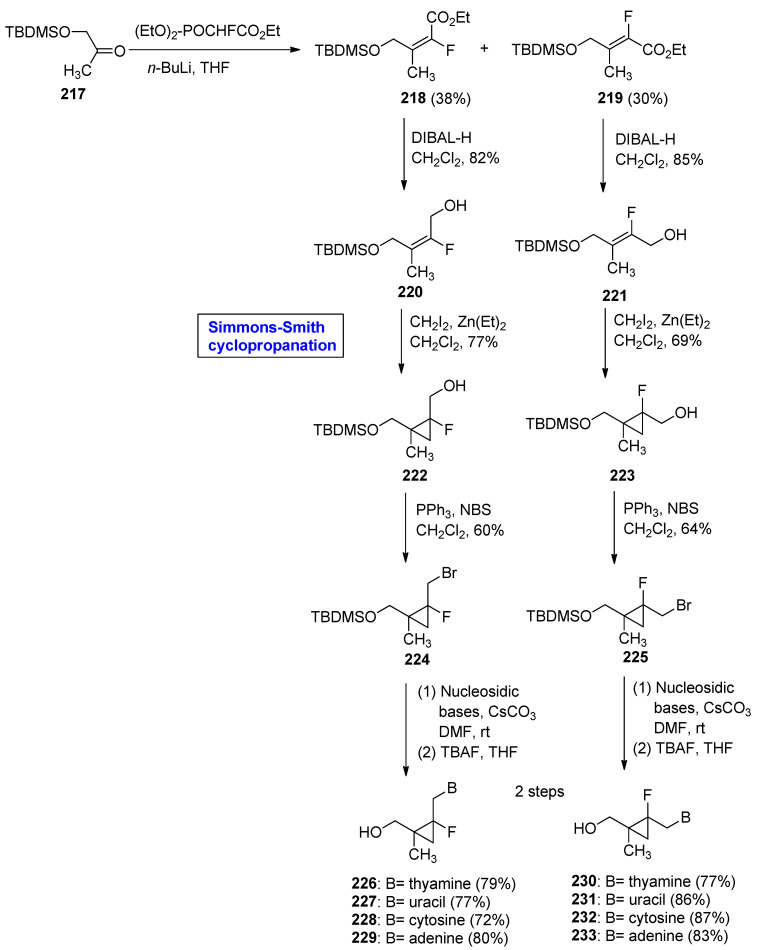
Synthesis of C-fluoro-branched cyclopropyl nucleosides (**226**–**233**).

The introduction of a cyclopropyl ring within nucleosides is the source of improving their antiviral activities [114]. Pioneering the antiviral studies of nucleoside derivatives in 2007, Kim and Hong synthesized C-1 or C-3 fluoro-substituted cyclopropyl rings containing nucleosides and evaluated their antiviral activities [115]. The synthesis involved Simmons–Smith cyclopropanation as the main step. In their methodology, readily available starting material acetal **217** was treated with (EtO)_2_-POCHFCO_2_Et in the presence of butyl lithium and THF to produce fluoroesters **218** and **219** in 38% and 30% yields, respectively. In the next step, reduction in carbonyl functionality was undertaken by using DIBAL-H in the presence of CH_2_Cl_2_ to produce fluoro-substituted allylic alcohols **220** and **221** in 82% and 85% yields, respectively. After this, cyclopropanation was performed by using the Simmons–Smith protocol in the presence of ZnEt_2_ and CH_2_Cl_2_, resulting in compounds **222** and **223** in 77% and 69% yields, respectively. In the next step, a nucleophilic substitution reaction was performed by using PPh_3_ and NBS to produce compounds **224** and **225** in high yield. In the last two steps, condensation of compounds **224** and **225** with nucleosidic bases in the presence of cesium carbonate and DMF followed by deprotection (in the presence of TBDMS and THF) successfully furnished the desired nucleosides **226** to **233** in high yield (Scheme 31). The synthesized compounds showed anti-HCMV activity. Uracil derivative **227** showed the most potent activity with EC_50_ = 10.61 μg/mL (Scheme 31).

**Scheme 32:**
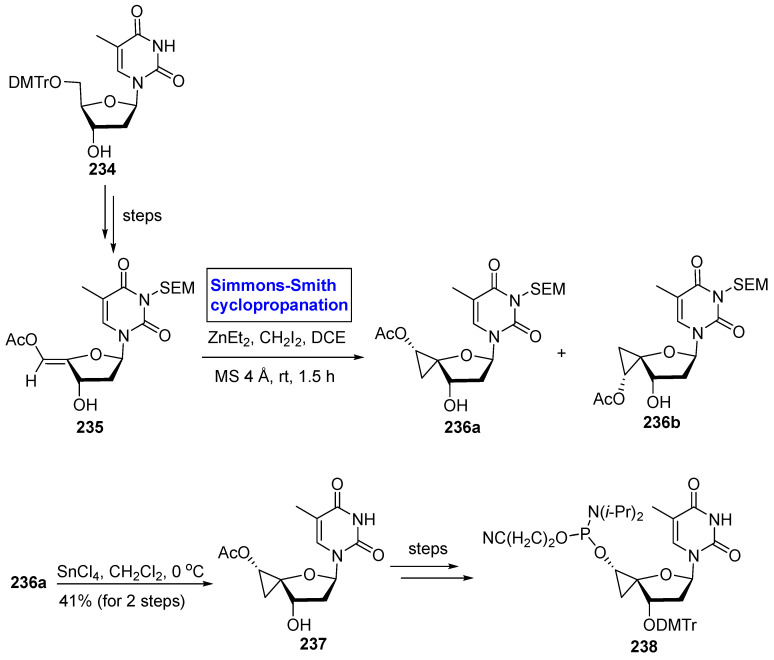
Synthesis of 4′,5′-BNA phosphoramidite **238**.

Oligonucleotides are renowned for their use as therapeutic agents against various diseases. The functions of oligonucleotides depend directly on the conformation and structural arrangement of their sugar molecules [116]. The role of tricyclo-DNA-based oligonucleotides for the treatment of Huntington’s disease and Duchenne muscular dystrophy is also being explored [117,118]. Considering the importance of oligonucleotides, Yamaguchi et al. [119] in 2021 performed the synthesis of 4′,5′ -BNA phosphoramidite **238** in 11 steps and incorporated it into oligonucleotides and evaluated its duplex-forming ability with RNA and DNA. In their methodology, thyamine **234** (as a starting material) was modified to produce compound **235** over a few steps. Simmons–Smith cyclopropanation of compound **235** in the presence of diethyl zinc and diiodomethane, at room temperature, resulted in the diastereomeric mixture of **236a** and **236b** (in dr = 10:3). Subsequent deprotection was performed to produce compound **237**. Compound **237** was reacted over a number of steps to successfully furnish 4′,5′-BNA phosphoramidite **238** (Scheme 32).

### 2.5. Synthesis of γ-Pyrone-Based Natural Product

**Scheme 33:**
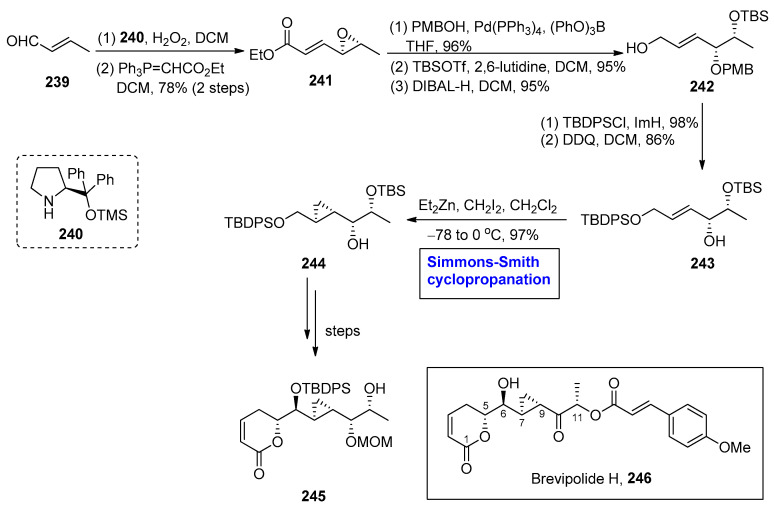
Synthesis of C1 to C12 fragment **245** of brevipolide H **246**.

Brevipolides A–F were first isolated from the plants of *Hyptis brevipes* Douglas Kinghorn in **2009.** These are famous for exhibiting antifungal, antibacterial and anticancer activities. Brevipolide H **246**, in particular, was isolated from *Lippia alva* (a Peruvian plant) [120]. It shows anti-HIV activity and possesses an attractive biological profile, which prompted researchers toward its total synthesis. The Hou group reported the enantiomeric synthesis of brevipolide H **246** [121]. In 2015, Mohapatra et al. disclosed the diastereoselective synthesis of the C1 to C15 skeleton of brevipolide H **246** via the readily available trans-crotonaldehyde **239** over 18 steps in a 12.5% overall yield [122]. The main steps entailed asymmetric Jorgensen’s epoxidation, the Pd-catalyzed opening of epoxide, Simmons–Smith cyclopropanation, Mitsunobu reaction [104], Brown allylation and Grubb’s catalyzed metathesis. Their synthesis commenced with epoxidation of trans-crotonaldehyde **239** in the presence of chiral catalyst **240** followed by Wittig reaction to produce epoxide **241** in a 78% yield with excellent diastereoselectivity and enantioselectivity (dr = 95:5 & ee = 93:7). The palladium-catalyzed ring opening of compound **241**, followed by TBS protection and subsequent reduction provided primary alcohol **242** (in a 95% yield), which was processed further by TBDPS protection and treatment with DDQ to produce allylic alcohol **243**. In order to install a cyclopropane ring, a well-suited Simmons–Smith protocol was applied in the following steps of adding diethyl zinc, diiodomethane and dichloromethane at −78 °C and slightly increasing the temperature up to 0 °C, successfully generating compound **244** in a 97% yield as a single diastereomer. By reacting compound **244** over a few steps, the synthesis of the desired fragment **245** was achieved in good yield (Scheme 33).

### 2.6. Synthesis of Polyketide-Based Natural Product

**Scheme 34:**
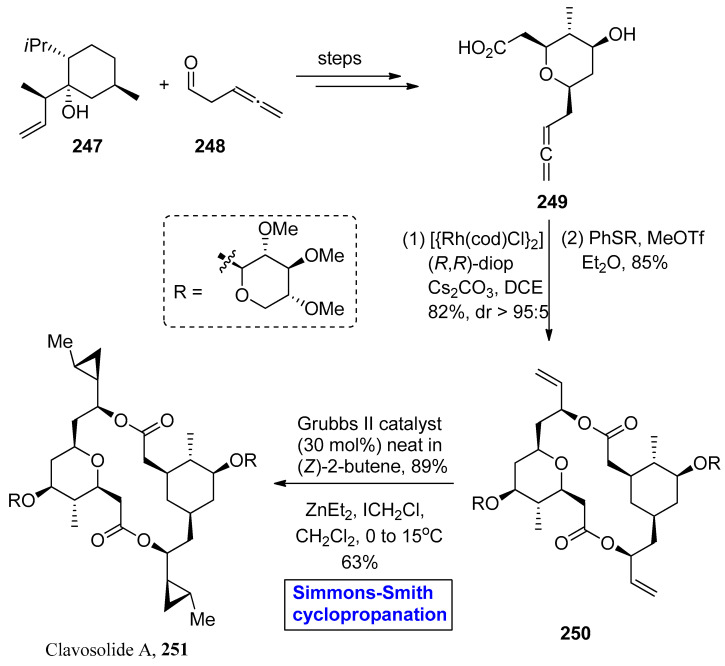
Synthesis of clavosolide A **251**.

Clavosolide A **251** belongs to the class of polyketides. Polyketides are natural metabolites, having large structural diversity and being renowned for exhibiting a large number of biological activities. Various synthetic strategies have previously been employed for the synthesis of the core structure of these metabolites, involving double Sakurai allylation [123], Mitsunobu reaction [104] and alkyne metathesis [124,125], but these strategies produced a large amount of waste and were not considered appropriate with respect to atom economy. In 2015, Haydl and Breit introduced a new, atom-economical, regioselective strategical procedure for the synthesis of a 16-membered skeleton of clavosolide A in eight steps [126]. The key steps involved the rhodium-catalyzed addition of carboxylic acid to alkene, cross metathesis and late-stage Simmons–Smith cyclopropanation. Compounds **247** and **248** were reacted over a few steps to produce allenyl-substituted carboxylic acid **249**. Fragment **300** underwent head-to-tail rhodium-catalyzed dimerization to produce compound **250** in an 82% yield with good diastereoselectivity. In the next step, cross metathesis of compound **250** was performed in the presence of Grubbs catalyst in (*Z*)-butene to produce an intermediate in an 89% yield (*E*/*Z* = 83/17), which was processed by Simmons–Smith cyclopropanation in the presence of diethyl zinc, ICH_2_Cl and CH_2_Cl_2_ to result in the successful synthesis of clavosolide A **251** in a 63% yield (Scheme 34).

### 2.7. Synthesis of Fatty-Acid-Based Natural Products

**Scheme 35:**
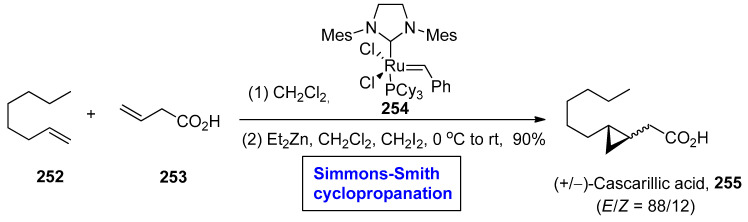
Total synthesis of (+/−)-cascarillic acid **255**.

Cascarillic acid **255** or grenadamide **258** are cyclopropane rings containing natural metabolites. Cascarillic acid is isolated from cascarilla essential oil, while grenadamide is isolated from marine cyanobacterium *Lyngbia majuscula.* In 2007, Salim and Piva performed the enantiomeric synthesis of cascarillic acid **255** and grenadamide **258** by employing cross metathesis and Simmons–Smith cyclopropanation as key steps [127]. In their methodology, a one-pot synthetic procedure was adopted, which proved to be time saving and effective in terms of obtaining good yield. For the synthesis of cascarillic acid **255**, a readily available starting material, i.e., vinyl acetic acid **253,** was allowed to react with 1-octene **252** in the presence of a ruthenium catalyst and dichloromethane as a solvent. The reaction mixture was heated for 24 h and then cooled to 0 °C. After that, diethyl zinc and diiodomethane was added in the same flask and kept stirred for 5 h, which resulted in a diastereomeric mixture of the desired product **255** in a 90% yield (after chromatographic separation) with *E*/*Z* = 88/12 (Scheme 35).

**Scheme 36:**
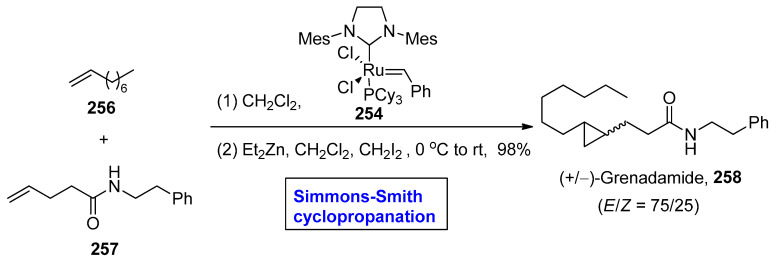
Total synthesis of (+/−)-grenadamide **258**.

The same procedure was adopted for the total synthesis of (+/−)-grenadamide **258**, in which amide **257** was allowed to react with 1-nonene **256** in the presence of ruthenium catalyst **254**. After cross metathesis, sequential Simmons–Smith cyclopropanation was employed by adding diethyl zinc and diiodomethane in the same pot at 0 °C, which furnished the mixture of **258** in a 98% yield with *E*/*Z* = 75/25 (Scheme 36).

**Scheme 37:**
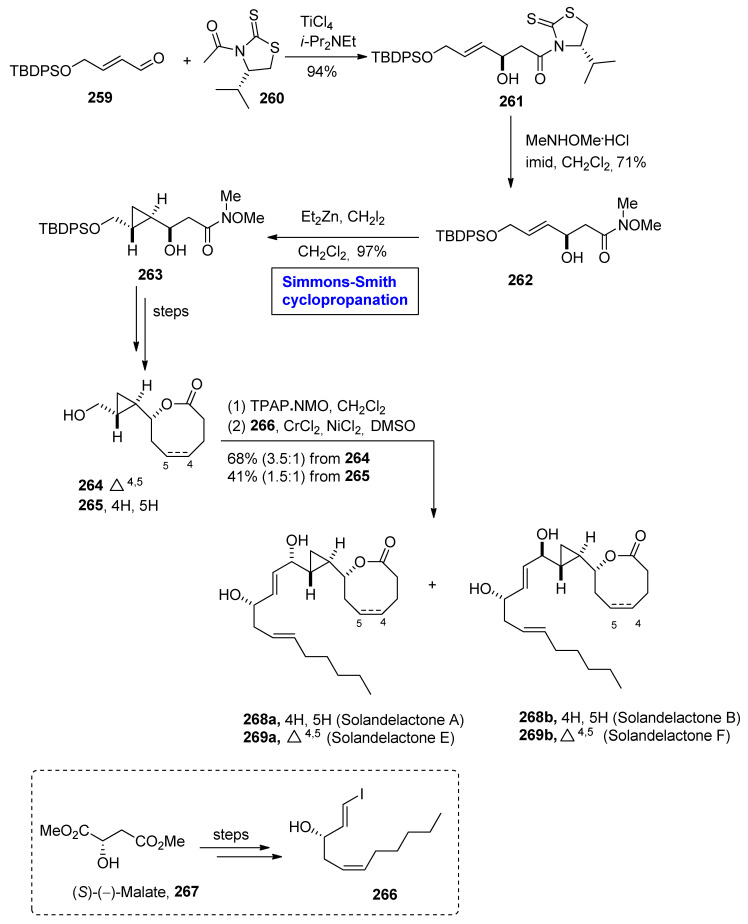
Synthesis of solandelactones A, B, E and F (**268a**–**269b**).

**Scheme 38:**
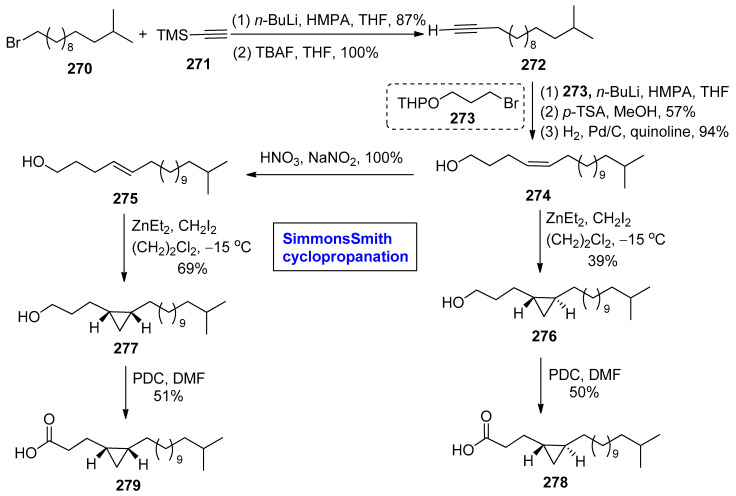
Synthesis of compound **278** and compound **279**.

Solandelactones A–H are complex marine fatty-acid metabolites that were first discovered by Shin and his colleagues in 1996. These were isolated from the *Solanderia secunda* present on Jaeju Island, Korea. Solandelactones A–H are also referred to as oxylipins. These metabolites have unique structural features with cyclopropane rings at C-9 and C-10. Different members of this class show diversity in their configurational behaviors, which makes their synthesis challenging and curious. According to Shin’s structural elucidation, the C-11 configuration of solendelactones A, C, E and G is assigned as *R*, while the C-11 configuration of solendelactones B, D, F and H is *S* [128]. In 2008, White et al. reported the synthesis of solandelactones A, B, E and F (**268a**–**269b**) by adopting a concise and efficient route [129]. Their methodology commenced with the Nozaki–Hiyama–Kishi coupling of aldehyde **259** with compound **260** in the presence of titanium chloride to yield compound **261** in a 94% yield. The treatment of hydroxyl amine **261** with *N*, *O*-dimethylhydroxylamine yielded compound **262**. After that, the hydroxyl-directed Simmons–Smith cyclopropanation was performed by adding diethyl zinc and diiodomethane in the presence of dichloromethane to produce compound **263** in a 97% yield with perfect control of stereochemistry. A few steps later, compounds **264** and **265** were allowed to react with aldehyde **266** in the presence of chromium chloride and nickel chloride in DMSO, which resulted in a stereoisomeric mixture of solandelactone A **268a** and solandelactone B **268b** in 3.5:1 from **264** in a 68% yield, as well as solandelactone E **269a** and solandelactone F **269b** in 1.5:1 from **265** in a 41% yield, respectively (Scheme 37). The stereochemical confirmation of the synthesized derivatives was confirmed by NMR spectroscopy, and the results showed that the C-11 configuration of the synthesized derivatives was opposite to the configuration that was actually assigned by Shin.

(±)-17-methyl-trans-4,5-methyleneoctadecanoic acid **278** is a marine cyclopropane fatty acid. It was first isolated from *Pseudospongosorites suberitoides*, a Caribbean sponge [130]. In 2010, Carballeira et al. performed the first total synthesis of (±)-17-methyl-trans-4,5-methyleneoctadecanoic acid **278** and its analogue, (±)-17-methyl-cis-4,5-methyleneoctadecanoic acid **279**, in eight steps (9.1% overall yield) and seven steps (16.4% overall yield), respectively, by employing Simmons–Smith cyclopropanation as a key step. 1-Bromo-12-methyltridecane **270** was used as the starting material, and both the synthesized isomers were evaluated for anti-leishmanial activity [131]. In the first step of their synthetic route, 1-bromo-12-methyltridecane **270** was allowed to react with trimethylsilyl acetylene **271** in the presence of *n*-BuLi and subsequently desilylated to produce 14-methylpentadec-1-yne **272** (in a 100% yield), which was processed by reaction with **273**, followed by deprotection by using *p*-TSA and subsequent reduction, to furnish compound **274** in a 94% yield. For the synthesis of **278**, cyclopropanation of compound **274** was performed by adding well-suited Simmons–Smith reagent, i.e., diethyl zinc, diiodomethane and 1,2-dichloroethane, as a solvent to furnish compound **276** (in a 38% yield). Compound **276** was processed further for oxidation via pyridinium dichromate to produce the desired product **278** in a 50% yield. In another route, the compound **274** was treated with nitric acid and sodium nitrate to produce compound **275**, which was then cyclopropanated by providing the Simmons–Smith conditions to produce compound **277** in a 69% yield. In the final step, oxidation of compound **277** furnished compound **279** (Scheme 38).

### 2.8. Synthesis of Drugs

**Scheme 39:**
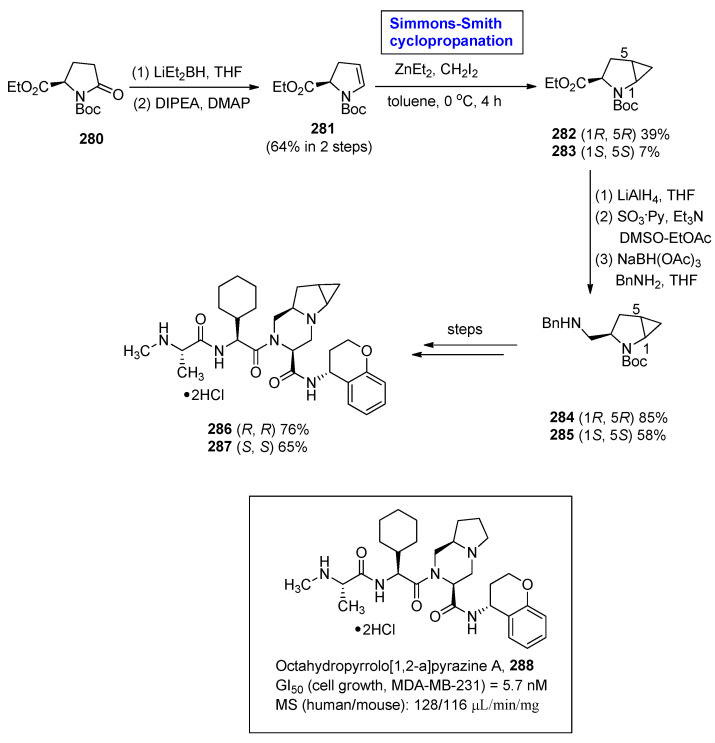
Synthesis of octahydropyrrolo[1,2-a]pyrazine A derivatives **286** and **287**.

Octahydropyrrolo[1,2-a]pyrazine A **288** is an effective anticancer agent, as it is an IAP (inhibitor of apoptosis proteins) antagonist. IAPs cause resistance to several chemotherapeutic drugs [132]. The tricyclic framework of octahydropyrrolo[1,2-a]pyrazine A has the ability to interact with IAPs via van der Waal forces, which promote cell death. However, octahydropyrrolo[1,2-a]pyrazine A **288** was found to be metabolically less stable [133]. The interesting biomimetic behavior of this heterocyclic scaffold prompted synthetic efforts toward the synthesis of its derivatives with improved metabolic stability. In 2013, Asano et al. disclosed the enantioselective synthesis of a cyclopropane ring containing derivatives of octahydropyrrolo[1,2-a]pyrazine A with an improved PK profile and better cytotoxic activities against cancer cells [134]. Their methodology commenced with the synthesis of the methyl ester derivative **281** of proline from the readily available starting material **280**. In order to install a cyclopropane ring, compound **281** was treated with Simmons–Smith conditions, i.e., diethyl zinc and dichloromethane in the presence of toluene. The temperature was initially kept at 0 °C and then raised slowly to room temperature, resulting in a diastereomeric mixture of compounds **282** and **283** in 39% and 7% yields, respectively (after chromatographic separation). After that, **282** and **283** were reduced in the presence of lithium aluminum hydride and subsequent oxidation in the presence of sulfur trioxide and pyridine complex, followed by reduction with benzyl amine, resulting in intermediates **284** and **285** in 85% and 68% yields, respectively. Over a few steps, intermediates **284** and **285** were converted into octahydro-1H-cyclopropa[4,5]pyrrolo[1,2a]pyrazine derivatives **286** and **287** in quantitative yields (Scheme 39). Both of the synthesized derivatives showed better metabolic stability. Moreover, derivative **287** exhibited antiproliferative activity against human breast cancer cells.

**Scheme 40:**
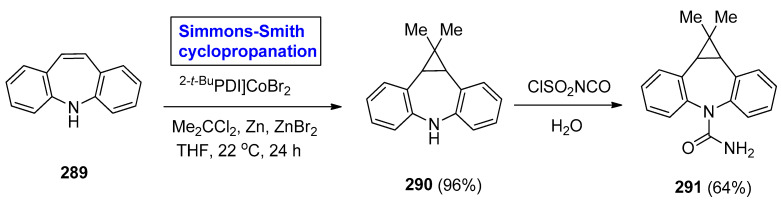
Synthesis of carbamazepine analogue **291**.

Carbamazepine analogues belong to the class of tricyclic antidepressants (TCAs) and are used in the treatment of neuropathic pain and epilepsy [135,136]. Werth and Ueyda [137] in 2018 performed a single step, highly regioselective synthesis of carbamazepine analogue from parent 5*H*-dibenz-s[b,f]azepine **289** by employing cobalt-catalyzed Simmons–Smith conditions. Compound **289** was treated with 10 mol% of [^2-*t*-Bu^PDI]CoBr_2_, 0.14 mmol of 1,3-diene, 2 eq. of Me_2_CCl_2_, 2 eq. of Zn and 1 eq. ZnBr_2_ to produce compound **290** with a 96% yield. In the next step, derivatization of compound **290** was performed by using chlorosulfonyl isocyanate, furnishing compound **291** in a 64% yield (Scheme 40).

**Scheme 41:**
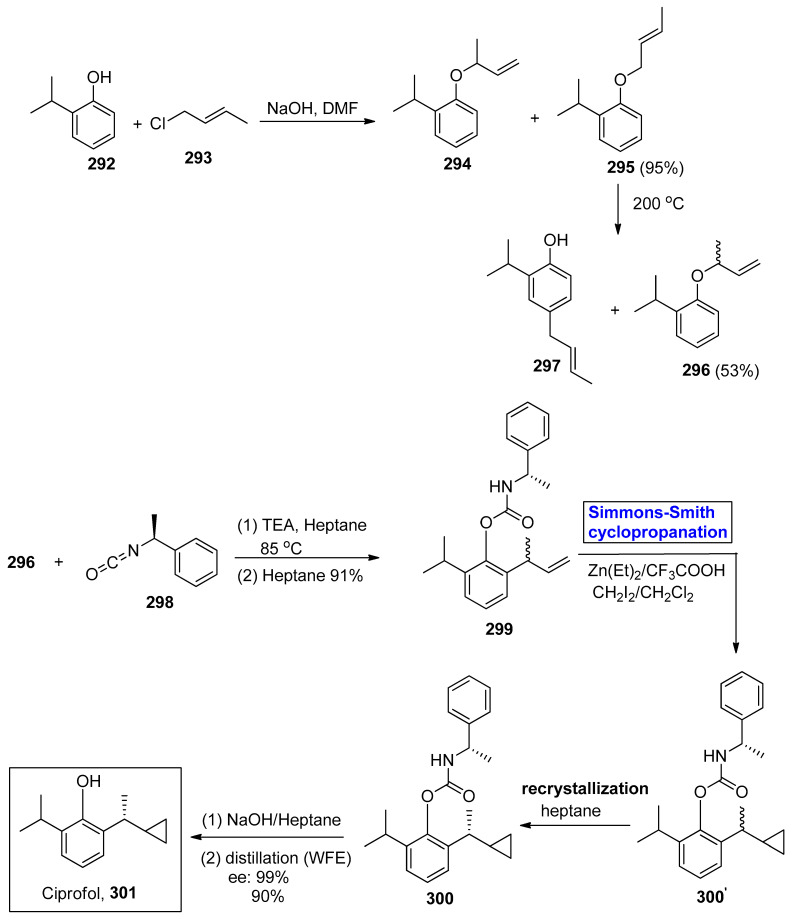
Kilogram-scale synthesis of ciprofol **301**.

**Scheme 42:**
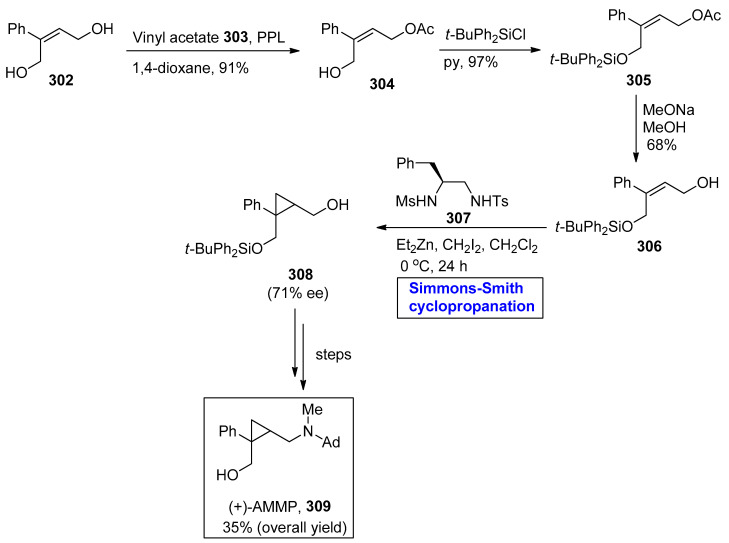
Synthesis of (+)-AMMP **309**.

Profol is a GABA_A_ (γ-aminobutyric acid) receptor agonist, widely used as an anesthesia and in the treatment of many psychological diseases. Ciprofol is also a GABA_A_ receptor of equal importance, having the fewest side effects (such as low blood pressure or respiratory depression) [138,139]. Zhang et al. [140] in 2022 evaluated and optimized the kilogram-scale route for the synthesis of Ciprofol, with 12–14% overall yield. The first route that they adopted for the synthesis faced some limitations, such as the use of organometallic reagents that caused toxic impurities and low-yield problems. The second-generation route was based on five steps by utilizing easily available starting material such as 2-isopropylphenol **292**. The treatment of phenol **292** with 3-chlorobutene **293** in the presence of NaOH as a base and DMF as a solvent resulted in a stereoisomeric mixture of compounds **254** and **253**. After that, compound **295** was separated from the unwanted compound **294** by column chromatography in a 95% yield and underwent Claisen rearrangement to produce an isomeric mixture of compound **296** and unwanted compound **297**. After chromatographic separation, compound **296** was obtained in a 53% yield. In the next step, compound **296** was allowed to react with compound phenyl ethyl isocyanate **298** in the presence of heptane to produce carbamate **299** in an excellent yield (91%). The Simmons–Smith cyclopropanation of compound **299** in the presence of diethyl zinc, CF_3_COOH and CH_2_I_2_ in 3:4:3 (optimized equivalents) resulted in racemic product **300^/^** (99% conversion). In the next step, recrystallization was performed to produce the desired product **300** with a 30 to 35% yield and a diastereomeric excess greater than 90%, followed by subsequent hydrolysis in the presence of NaOH and heptane, and then finally, distillation produced the desired ciprofol **301** (Scheme 41).

(+)-Cis-4-(*N*-adamantyl-*N*-methylamino)-2,3-methano-2-phenylbutan-1-ol (+)-AMMP) **309** is a sigma receptor agonist. Sigma agonists control numerous cognitive brain functions to prevent dementia and memory-loss problems. Therefore, (+)-AMMP **309** is expected to be an interesting candidate for the treatment of Alzheimer’s disease [141]. The synthesis of (+)-AMMP **309** has been reported in the literature by using (+)-2,3-methano 2-phenyllactone, an expensive starting material, and only a 9% yield was achieved [142]. As a part of ongoing research, Kawahima et al. [143], in 2016, performed the enantioselective synthesis of compound **309** with a 35% overall yield by using readily available starting material and cheap reagents. The key steps entail PPL-induced acetylation, catalytic Simmons–Smith cyclopropanation and amidation. In the first step of their synthetic route, (*Z*)-3-phenylbut-2-en-1,4-diol **302** was protected in the presence of vinyl acetate **303** and 1,4 dioxane to yield compound **304** with a 91% yield. Then, silyl protection of compound **304** in the presence of pyridine produced compound **305** in a 97% yield, followed by subsequent acetyl deprotection in the presence of MeONa and MeOH to produce compound **306** in a 68% yield. In the next step, compound **306** underwent catalytic Simmons–Smith cyclopropanation by using two equivalents of diethyl zinc, three equivalents diiodomethane and one equivalent of compound **307** as a catalyst at 0 °C, furnishing compound **308** in a quantitative yield and 71% ee. A few steps completed the synthesis of (+)-AMMP **309** with a 35% overall yield (Scheme 42).

**Scheme 43:**
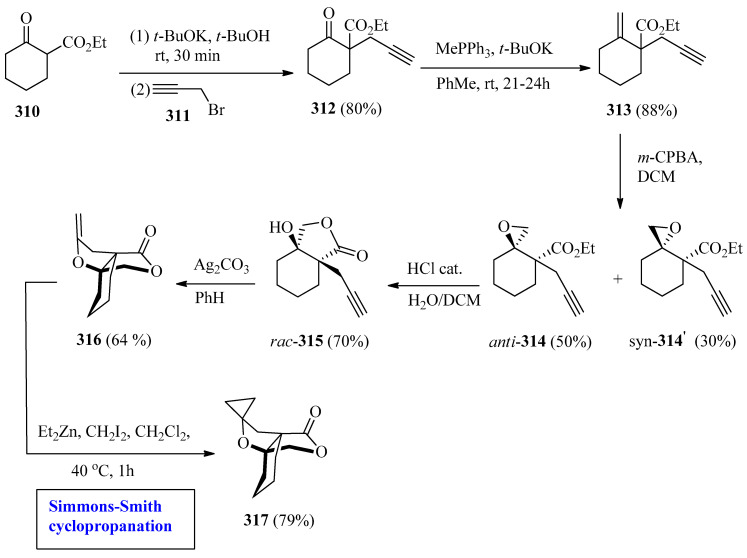
Synthesis of oxaspiro[n,3,3]propellane **317**.

Oxaspiro[n,3,3]propellanes are attractive heterocyclic scaffolds in medicinal chemistry. Their tricyclic structure is present in many natural compounds such as marasmic acid, modhephene and bukittinggine. Many synthetic attempts have been made for the synthesis of oxaspiro[n,3,3]propellanes, but it was still a challenge for many researchers [144,145]. Nassar and Piva [146] in 2021 presented a valuable synthetic route toward the synthesis of oxaspiro[n,3,3]propellane (**317**). The key steps involved hydroxymethylation (either photochemical or alternative routes consisting of three steps), metal-catalyzed cyclization and Simmons–Smith cyclopropanation. For the synthesis of **315** in high yield, a synthetic route was adopted in which compound **310** was allowed to react with propargyl bromide **311** in the presence of a base under reflux for 4 h to produce compound **312** in 95% and 80% yields, respectively. In the next step, a Wittig reaction was performed to produce compound **313**, followed by oxidation in the presence of *m*-CPBA and dichloromethane, which furnished an inseparable mixture of diastereomers (**314** and **314^/^**). Subsequently, compound **314** was treated with HCl, water and DCM to furnish the bicyclic lactone **315**. Later, silver-catalyzed cyclization of compound **315** in the presence of benzene at 60 °C yielded compound **316** in a 64% yield. In the last step, the Simmons–Smith cyclopropanation of compound **316** in the presence of diethylzinc and diiodomethane at 40 °C, under reflux condition, furnished our desired compound **317** in a 79% yield (Scheme 43).

**Scheme 44:**
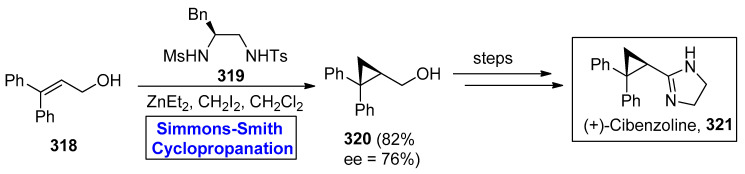
Synthesis of (+)-cibenzoline **321**.

Cibenzoline **321** is an anti-arrhythmic drug. Its structure contains two benzene rings, one imidazoline ring and a cyclopropane ring with a stereogenic carbon [147]. Considering its biological importance, Miura et al. [148] in 2006 performed the synthesis of cibenzoline and its analogues by employing sulfonamide-catalyzed enantioselective Simmons–Smith cyclopropanation. In their methodology, 3,3-diphenyl-2-propen-1-ols **318** was treated with diethyl zinc, diiodomethane and dichloromethane in the presence of a catalytic amount of (*S*)-phenylalanine-derived disulfonamide **319** to furnish cyclopropylmethanol **320** in an 82% yield with 76% enantioselectivity. Over a few steps, compound **320** was modified into (+)-cibenzoline **321** with a 55% yield (Scheme 44).

**Scheme 45:**
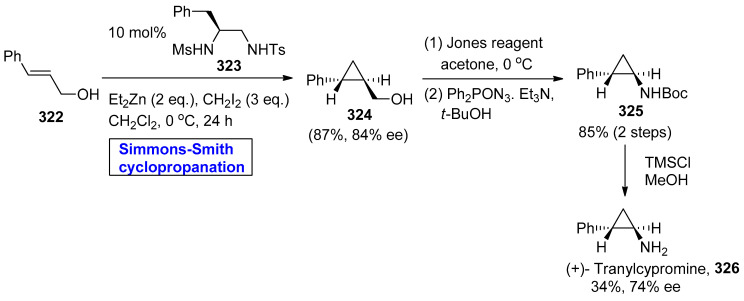
Synthesis of (+)-tranylcypromine **326**.

Tranylcypromine and milnacipran (cyclopropane-based amines) are strong antidepressants. Tranylcypromine is also an anxiolytic agent, thus is effective for the treatment of anxiety and mood disorders [149]. Milnacipran is a serotonin and norepinephrine reuptake inhibitor (SNRI). The stereospecific analogues of both these compounds are expected to show more interesting biological and therapeutic activities [150]. In 2013, Ishizuka et al. performed the asymmetric synthesis of (+)-tranylcypromine **326** (in a 22% overall yield) and (−)-milnacipran hydrochloride **331** (in a 14% overall yield) by employing sulfonamide-catalyzed enantioselective Simmons–Smith cyclopropanation as a key step [151]. For the synthesis of (+)-tranylcypromine **326**, trans-cinnamyl alcohol **322** (as a starting material) was treated with two equivalents of diethyl zinc, three equivalents of diiodomethane in the presence of sulfonamide catalyst **323** and diiodomethane as a solvent at 0 °C to furnish compound **324** in an 87% yield with 84% ee. After asymmetric sulfonamide Simmons–Smith cyclopropanation, compound **324** was oxidized by using Jones reagent, followed by treatment with diphenylphosphoryl azide to furnish compound **325**. The deprotection of compound **325** in the presence of TMSCl resulted in the final product **326** in a 34% yield with 74% ee (Scheme 45).

**Scheme 46:**
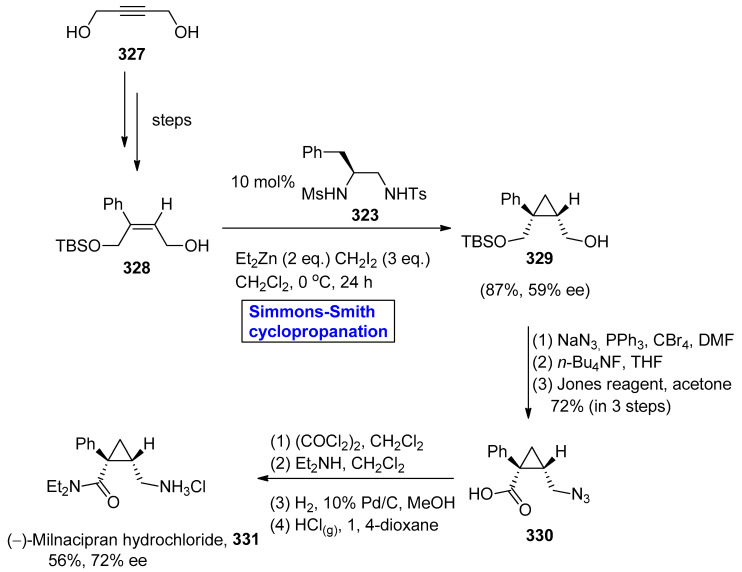
Synthesis of (−)-milnacipran hydrochloride **331**.

The synthesis of (−)-milnacipran hydrochloride **331** was undertaken by using diol **327** as a starting material. After modification of compound **327** over a few steps, the asymmetric sulfonamide Simmons–Smith cyclopropanation of **328** was performed under the previously mentioned conditions (as for (+)-tranylcypromine **326**) to furnish compound **329** in an 87% yield (with 59% ee). The OH group of **329** was converted into the azide, followed by deprotection with tetra butyl ammonium fluoride and subsequent Jones oxidation resulting in compound **330** in a 72% yield. In the next step, the carboxylic functionality of compound **330** was transformed into amide and processed by hydrogenation and subsequent treatment with HCl to produce the desired final product **331** in a 56% yield with 72% ee (Scheme 46).

## 3. Conclusions

The concise and stereospecific synthesis of cyclopropane-based natural products with exact configuration of their stereogenic centers by employing Simmons–Smith cyclopropanation has been highlighted throughout this review. Previous strategies for Simmons–Smith cyclopropanation were limited to the use of zinc metal along with diiodomethane. With the passage of time, various modifications have been made and reported in the Simmons–Smith reagent, such as the Denmark modification (Et_2_Zn and ClCH_2_Cl), Furukawa modification (Et_2_Zn and CH_2_Cl_2_) and Charette modification (bipy. Zn(CH_2_I)_2_ complex). However, the synthetic strategies discussed in this review are based on the Furukawa modification (Et_2_Zn and CH_2_Cl_2_). Furukawa-modified Simmons–Smith cyclopropanation is efficient and is the most preferred strategy in modern organic synthesis, as it retains all characteristics (that are present in classical ones) and can be applied over a wide range of temperature. The panoramic features of this reaction entail a broad range of substrate compatibility, hydroxyl-substituted directing effect, good enantiocontrol, minimum handling and purification difficulties, maximum yield and the generation of a complex molecular architecture with desired stereochemistry. The enantioselective synthesis of some polycyclic structures has also been reported by employing Simmons–Smith cyclopropanation via a one-pot synthetic strategy. Although much effort has been put into methodology development, as well as the synthetic applications of Simmons–Smith cyclopropanation, by various research groups, the authors nevertheless believe that there is still a great vacuum in its applications toward pharmaceutically important molecules. Moreover, the Simmons–Smith-cyclopropanation-based synthetic schemes discussed herein would open up new routes toward the synthesis of novel heterocyclic compounds in medicinal chemistry.

## Data Availability

All data are contained in the manuscript.
